# Belief in a Just What? Demystifying Just World Beliefs by Distinguishing Sources of Justice

**DOI:** 10.1371/journal.pone.0120145

**Published:** 2015-03-24

**Authors:** Katherine Stroebe, Tom Postmes, Susanne Täuber, Alwin Stegeman, Melissa-Sue John

**Affiliations:** 1 Department of Social Psychology, University of Groningen, Groningen, The Netherlands; 2 Department of Human Resource Management & Organizational Behaviour, University of Groningen, Groningen, The Netherlands; 3 Department of Psychometrics & Statistics, University of Groningen, Groningen, The Netherlands; 4 Department of Social Science and Policy Studies, Worcester Polytechnic Institute, Worcester, Massachusetts, United States of America; Tilburg University, NETHERLANDS

## Abstract

People’s Belief in a Just World (BJW) plays an important role in coping with misfortune and unfairness. This paper demonstrates that understanding of the BJW concept, and its consequences for behavior, is enhanced if we specify what (or who) the source of justice might be. We introduce a new scale, the 5-Dimensional Belief in a Just Treatment Scale (BJT5), which distinguishes five causal dimensions of BJW (God, Nature, Other People, Self, Chance). We confirm the 5-factor structure of the BJT5. We then address whether the BJW should be considered a uni- and/or multi-dimensional construct and find support for our multi-dimensional approach. Finally, we demonstrate convergent and discriminant validity with respect to important correlates of BJW as well as action in response to important negative life events and societal attitudes. This work illustrates the importance of distinguishing causal dimensions with regard to who distributes justice.

## Introduction

There is a basic tendency to believe that the world treats people more (or less) justly. According to theory, such a Belief in a Just World (BJW) enables people to see the world as a stable, orderly and safe place [1]). The BJW concept, originally proposed by Lerner [1, 2], has triggered a formidable line of research that clearly attests to its psychological relevance. Since the origin of the BJW concept a substantial literature has focused on how to best operationalize such beliefs [1–4]. Research has shown that it is important to distinguish the *target* of justice [5, 6]: There are important differences in believing that the world is just for the self versus for other people (the question of ‘justice *for* whom?’). Yet, so far research has not considered who or what is the source of this just world (the question of ‘justice *from* whom/what?’). The present work focuses on this question and argues that specifying who or what determines a just or unjust “world” is essential in understanding the BJW concept and its implications for behavior.

We propose that people’s coping with major life events and their emotional well-being is determined by the sources individuals see as being responsible for justice. Early work by Lerner [2] already pointed to different ways in which people can make sense of major life event, such as becoming paraplegic. People’s reactions to these events varied depending on whether they saw God, themselves, or Chance as responsible for this outcome. Moreover, people may try to make sense of such events by fitting them into their own frameworks of justice (e.g., seeing oneself as deserving a chance event such as the sudden death of one’s child; [7, 8]). In the present paper, we put forth the idea that people’s coping with major life events and their emotional well-being is determined by the sources individuals see as being responsible for justice. In other words, we expect that the actions people take in response to life events will match the source they hold responsible for the event. For example, seeing God as an important actor in life is associated with more positive psychological well-being, an ability to give meaning to negative life events, and a greater likelihood of prosocial behavior (e.g., [9, 10]). By contrast, attributing important specific life events as due to chance can be associated with negative well-being and feelings of loss of control (e.g.,[11, 12]). Thus, there are sound theoretical reasons why differentiating the source of justice is an essential requirement—one that is not fulfilled in the present conceptualization of a just “world” that gives people what they deserve.

We therefore developed and tested a new scale, the 5-Dimensional Belief in a Just Treatment Scale (BJT5), which distinguishes among five causal forces that may determine whether people get what they deserve in their lives: Nature, God, Other People, Self, and Chance. One of the aims of this research is to examine the psychological foundations of the BJW concept itself: Is BJW an overarching sense of justice that informs all individual justice perceptions within the BJT5 (e.g., BJW as one unitary construct or at least a general dimension)? Or is BJW a collection of individual justice beliefs, with each individual filling in the meaning of the concept “world” according to whatever she/he believes to be its most central elements (e.g., a world filled by Nature, God, Other People, Chance or the Self, depending on what is most salient in a particular context or valued by a particular individual)? A second aim is to show that this new scale provides more specific insight into the *different types of (in)action* individuals may take in response to injustice. And a final aim is to show how this refinement helps to clarify relations of BJW to particular correlates (e.g., religiosity; well-being) by tying them to concrete sources of justice.

### The Belief in a Just World

Lerner [2] describes the manner in which he began thinking about the BJW concept when he saw people working with dying children or being confronted with the bad health and suffering of parts of the American population. How do people function normally under, and cope with, extreme suffering? According to Lerner such injustices are particularly impactful because of people’s fundamental need to see the world as a place in which good deeds are rewarded and bad deeds are punished. Responses to such threats are motivated by the need to uphold one’s BJW: Individuals adapt their perceptions of injustice (or the reality at hand), for example by seeing unjust outcomes as caused by victims of injustice, rather than facing a potentially unjust world. This mechanism functions across many contexts (e.g., with respect to victims of AIDS, rape, robbery, cancer, discrimination; [1, 4, 13–16]). Building on this initial work (see also [4]), scales were developed to assess people’s explicit beliefs about the world as just. This research on scale construction reveals that individual differences in levels of BJW influence, among others, people’s goals in life [14], personal well-being [17–21], achievement behavior in schools [22], and responses to many types of personal and group disadvantage [23–28].

### Justice beliefs as a process: Who gives What to Whom?

Although the BJW as a unitary construct has proven predictive of responses to injustice [29–31], our understanding of the BJW concept itself still needs developing (e.g., [4, 5]). The supposed generic belief in a just world raises questions. What does it mean for “the world” to be just? A very basic approach would be to pare this down by treating justice as any other relational good. Who is the beneficiary of justice? What does just treatment entail? And who is the benefactor? Each of these questions offers scope for clarifying the BJW concept.

One important step in dissecting the BJW concept has been to distinguish among beneficiaries: Whether people are thinking about themselves or other people as the beneficiary of just treatment [6, 32–34]. This work shows that BJW for the self is more strongly related to psychological adjustment such as life satisfaction, lower levels of depression, and stress [6]. It buffers people from negative life events by providing a positive illusion of a stable and orderly world, even in the face of potential injustice. By contrast, the BJW for others may more strongly resemble the general motive to see the world as just and to see unjust outcomes as due to personal deservingness [32]. It is associated with negative societal attitudes towards lower status groups (e.g., the poor, the elderly) and perceptions of one’s social environment in general [21, 32, 35]. Yet, perceptions of justice may not only vary for self versus other, but also depend on the type of other, differing with respect to the specific referent group in question (e.g., seeing men as living in a more just world than women; [34]). With respect to the self-other distinction this provides a first indication of the need for multidimensionality, revealing that people make realistic adjustments to the perceived reality of the group they are assessing rather than engaging in overall broad justice judgments motivated by the desire to see the world as just for everyone. Thus, in a number of important ways, the self–other distinction has advanced our theoretical understanding of BJW and points to the benefits of specifying the BJW construct.

Other studies have suggested the value of considering multidimensionality in studying the Belief in a Just World construct. For example, the first BJW scale distinguished items referring to justice (e.g., people who do their job well rise to the top) and to injustice (e.g., good deeds often go unnoticed; [31]; see also [36, 37]). Others have distinguished justice with regard to the past/present (immanent justice) versus the future (ultimate justice; [38, 39]), or differentiated procedural and distributive justice [40, 41]. Finally, there have been attempts to distinguish justice domains (i.e., personal, interpersonal, socio-political; [42]). All these studies point to the different notions of justice that might comprise dimensions of the BJW construct [5].

#### Sources of justice: the BJT5 dimensions

In this paper we dissect the BJW concept quite differently from these prior attempts. We propose that a lot of additional understanding can be gained by differentiating among potential *sources* of (in)justice. In our view, the key concern with the BJW concept is that it does not specify what people mean when they endorse statements about “the world”. With the aim to specify this concept we developed the 5-Dimensional Belief in a Just Treatment Scale (BJT5) in which we distinguish between five factors that might determine justice outcomes in “the world”. These five important and causally influential sources of justice or injustice are Nature, God, Other People, Self, and Chance. We adjusted a validated and homogeneous BJW measure [30] so that items referred no longer to “the world” but to these more concrete sources (e.g., people get what they are entitled to have due to the forces of nature). We briefly outline why it is important to distinguish among sources of justice and then describe how we selected the five that form the dimensions of the BJT5.

Although intuitively it seems plausible that justice in the world could stem from numerous sources, the question of what/who is responsible for distributing it has received no prior attention in research. This is surprising given that there is reason to believe people are likely to consider who distributes justice. In the following, we will briefly review evidence from different lines of research supporting our argumentation in favor of a distinction between these five different sources of justice.

A number of different literatures provide support for the inclusion of these five dimensions. Studies looking at meaning giving after traumatic life events (such as the loss of a child) and measuring ultimate responsibility assigned for the cause of these events generally ask whether one considers oneself, another person, God, or chance responsible for this event (e.g., [43, 44]. These different attributions of causality are thought to affect how people cope with these events and whether and how people more generally cope with injustice and protect their BJW.

Other areas of literature (e.g., on personal control, determinism, religiosity; [45, 46]) provide additional reasons for considering sources of justice relevant in specifying responses to injustice. For example, Paulhus and Carey [47] distinguish four factors related to determinism: Free Will (corresponds to our Self dimension), Scientific Determinism (Nature dimension), Fatalistic Determinism, and Unpredictability (Chance dimension). Levenson and Miller [45], in studying a multidimensional model of control, refer to three potential dimensions: Self, Chance, and powerful others (Other People dimension).

Taken together the above reveal that based on quite distinct literatures we can derive five potential dimensions of justice that so far have not been considered jointly yet seem essential to people in understanding the world that surrounds them. They formed the basis for our new scale, the 5-Dimensional Belief in a Just Treatment Scale (BJT5), in which we distinguish among five causal forces which correspond to sources of justice people may deem important, influential, and ultimately responsible for outcomes in life: Nature, God, Other People, Self, and Chance. We outline these sources and their relevance below.

#### God

Religiosity has consistently correlated highly with the BJW [2, 6]. Indeed, attributing unjust and tragic outcomes in one’s own and the life of others to God’s will can be a way of coping with these negative life outcomes and of regaining feelings of personal control by attributing negative outcomes to God’s will [46]. In line with our idea that the perceived source of justice influences action tendencies, those who are religious experience more positive well-being/affect and engage in more prosocial behavior (e.g., [9, 10]).

#### Self, other people and chance

*Self*, *other people* and *chance* are considered central elements in determining perceptions of and responses to life events within the personal control, determinism, and attribution literature (e.g., [45, 47–51]). For example, research on personal control has distinguished *self* (or internal) from more ‘external’ forms of control such as powerful *other people* and *chance* (e.g., [45, 52]). In fact, this distinction between Self, Chance, and Other People was translated to the BJW area, but was psychometrically weak and focused on spheres of just/unjust outcomes rather than sources of justice [30, 42].

In some ways chance, being a more random source of justice than the other dimensions, might be seen as most weakly related to the concept of justice (see also Lerner, 1980). Yet there is reason to believe that people fit chance into their frameworks of justice. For one, different areas of literature reveal that people are able and motivated to make sense of chance events, for example by seeing chance events as deliberate, in a way that makes these chance events fit into their framework of justice [7, 53, 54]. Moreover, attributing a chance event to chance as a source of justice may be beneficial in meaning giving especially when events are perceived as very hopeless and out of control: In assessing interpretations of mothers whose children were undergoing severe bone marrow treatment, Rini and colleagues [8] found that mothers’ interpretations of the fate of their children as due to chance were positively correlated with adaptation. This implies that in some cases (random events) seeing chance as a source of justice may potentially help rather than hinder the ability to maintain the view that the world is essentially fair.

Importantly, the Chance, Self, and Other People sources of justice have also been shown to be essential in specifying levels of activism: The belief in powerful others is more consistently related to activism than Self and Chance [45, 55].

#### Nature

The concept of *nature* or *natural forces* incorporates the idea of a causal and powerful force that has far reaching consequences that are largely beyond human control. It is seen as one of the lay beliefs people can have about their outcomes in life within the determinism literature [47]. Importantly, an interpretation of outcomes as due to nature is consequential: In the context of disasters that are attributable to nature or other people, those attributable to nature have been shown to induce more positive and active responses (e.g., altruism, community support) than those attributable to other people [56, 57].

Although not directly related to the area of justice, these different literatures attest to the importance of distinguishing sources of justice in studying behavior. Yet, in contrast to our scale, none of these literatures cover all the sources outlined above and deemed important in understanding whom people see as the source of justice. Furthermore, compared to other scales of personal control or fate/determinism, our scale has the advantage of uniformity: we use the same set of items to address the different proposed dimensions.

#### Addressing injustice

We argue that providing individuals with specific types of actions that befit their predominant sources of (in)justice establishes the necessary conditions for taking action. Here we challenge recent research that looks at the relation between the BJW and more general forms of action. This literature reveals that BJW is related to lack of action among others because those who have a high BJW generally assume all will turn out well in the long run (e.g.,[23, 26]). Interestingly, the original BJW literature posits that individuals will act out against injustice when they have the means to do so (e.g., [1, 2]).

We argue that having to specify dimensions that are the ultimate cause of just/unjust treatment can provide a clearer potential to re-establish justice and take action for two reasons. Firstly, it makes salient to people the origins of and the responsibility for reinstating injustice/justice, countering the idea that all will turn out well in the long run—somehow (e.g., [26, 58]). Secondly, it provides individuals with a clear course of action. One cannot appeal to “the world”. But if one believes that there is a God who will ensure that justice is done, one has a very clear course of action: to pray or make offerings. Similarly, if other people are deemed responsible for justice in the world, one can appeal to them or their institutions. In studies looking at the BJW, the sources of justice are not specified but rather are reflected in a general notion of “the world”. Conversely, the types of potential action specified thus far in research may not map well onto the specific source beliefs and needs of individuals responding to injustice. This may well be the reason why BJW does not explain well what actions individuals take upon the injustice they encounter. Therefore we predict that, in contrast to a more general BJW measure, the BJT5 is likely to predict action.

Yet, in line with research on personal control discussed above, we propose that some sources of justice are more likely to be related to action than others: Action is more likely when BJW is seen as caused by God, Nature or Other People (e.g., [10, 45, 55]).

#### Conclusions

So far we have outlined 5 causal dimensions that form the basis of the BJT5 (Nature, God, Other People, Chance, the Self). This list of dimensions may be incomplete and specific to contemporary Western culture. The aim of the present study was to consider whether these five dimensions are indeed separate constructs that contribute to our understanding of the BJW. In other words, is the BJW an overarching sense of justice or, as the BJT5 implies, a collection of potentially distinct justice beliefs tied to specific sources (Nature, God, Other People, Chance, or the Self)? We do so by developing the BJT5 scale, considering whether it is a uni- and/or multidimensional construct, and whether it indeed differentially relates to important correlates of the BJW. Importantly, we also study the extent to which the BJT5 differentially colors perceptions of important (unjust) life events and societal attitudes and influences action versus inaction tendencies.

## Outline of the Studies and Participants

### Participants and Samples

We tested the BJT5 scale in four data sets (Sample 1, 2, 3 and 4). Sample 1 was composed partly of American undergraduate students from a Northeastern university (64 men, 48 women, 5 missing values) who completed this study in the laboratory, and partly of respondents recruited via Amazon’s MTurk who completed the study online (94 men, 152 women). Overall, 75% of participants were Caucasian, 6% African American, 8% Asian, and 11% of other ethnicity (*M*_*age*_ = 31; *SD*_*age*_ = 12.81). Of the 363 participants in total, 18 had missing data for the BJT5 scale and were excluded from analyses.

Sample 2 (3 men, 121 women; *M*_*age*_ = 34, *SD*_*age*_ = 13.43) completed the study online and partly consisted of undergraduate students (N = 11) and partly of respondents recruited via Amazon’s Mturk (N = 113). 80% of participants were Caucasian, 3% African American, 5% Asian, 7% of other ethnicity, and 5% did not indicate ethnicity. Four participants had missing data for the BJT scale and were excluded from analyses. This was (chronologically) the first study in the development of the BJT5, and at this time did not include the Chance dimension of the full scale. Therefore we refer to the BJT, (rather than the BJT5), when presenting Sample 2. All other dimensions were measured identically across studies. Originally, we included the dimension of BJW as determined by human institutions (e.g., government, companies) in both samples. In Sample 1 we found no distinction between the human institution and Other people dimension (i.e., they loaded on the same factor), in Sample 2 we did. As the concept Other people is broader and can be seen to encompass the human institution dimension, we decided to exclude the human institution dimension in this manuscript.

Sample 3 (35 men, 61 women, 4 unspecified; *M*_*age*_ = 21, *SD*_*age*_ = 3.18) completed the study online and consisted of international bachelor students of German (67%), Dutch (8%), and other nationalities (25%). Of the 100 participants in total, 8 had missing data on the BJT5 scale and were excluded from analyses.

Sample 4 (36 men, 70 women; *M*_*age*_ = 21, *SD*_*age*_ = 1.43) completed the study in the lab and consisted of international bachelor students of German nationality. This was part of a larger study looking at discrimination against German students.

### Ethics Statement

For Sample 1 we obtained ethical approval from the Ethical Committee Psychology of the University of Groningen for Mturk and from the IRB of Worcester Polytechnic Institute for the lab part of this study (IRB# 11-195 “Levels of Just World Beliefs: Scale Validation”). Samples 2, 3 and 4 were approved by the Ethical Committee Psychology of the University of Groningen. Across all samples written informed consent was obtained from all participants immediately before the research commenced.

### Outline of the studies

For reasons of clarity we outline the scale development of the BJT5 in analytical steps throughout the paper. Each of these steps reflects separate analyses conducted on one of the four samples. In step 1, we used a Principal Component Analysis to confirm the 5 factor structure of our scale (Sample 1, 3). In addition, we aimed at item reduction (Sample 1). Step 2 tested whether the BJT5 is better conceptualized as a uni- or multidimensional construct (Sample 1). In step 3 (Sample 1), we focus on convergent and discriminant validity with respect to important correlates of the BJW scale. Steps 4 (Sample 2) and 5 (Sample 1) assess relations between the BJT5 and behavioral measures in response to important negative life events. Step 6 (Sample 4) considers the relation between the BJT5 and attitudes towards criminal behavior in society.

## Steps 1 and 2: Participants, Materials and Procedure

We made use of Sample 1 for steps 1 and 2, and of Sample 3 for step 1. The 5-dimensional Belief in a Just Treatment scale was constructed by adapting the seven items from the Lipkus [6, 30] scales to specify 5 possible sources of just treatment (e.g., people get what they are entitled to have *from God*). Belief in a Just Treatment for Self and other People was assessed on scales from 1(strongly disagree) to 7 (strongly agree) by asking participants to indicate the extent to which “your own life”/”the life of other people” is affected by these sources. The order of the self and other-focused questions was counterbalanced.

## Step 1: Exploratory Factor Analyses and Item Reduction

### Initial Analyses

We combined lab and online data after several checks suggested they were similar enough to do so. Means and standard deviations for both populations were substantially the same. The correlations were quite similar and each dataset yielded similar factor scores. Details of these checks can be found in the online supporting information (S1 File). We also conducted Bonferroni corrected t-tests to examine whether order of questions in the questionnaire resulted in differences. There were no significant order effects. Finally, we checked normality of the items. For most of the subscales there were no issues, but items for God were slightly bimodal (resulting in negative kurtosis, −1.4). For Nature items, there was some kurtosis as well (−.78). Tests for multivariate normality suggested only minor deviations from normality.

### Factor Analyses of the BJT5 Scale

Items were factor analyzed separately for self and others, using Promax rotation. In view of the minor deviations from normality we used Principal Axis Factoring which is more robust [59]. We extracted factors with an eigenvalue greater than 1. Factor loadings, eigenvalues, and scale reliabilities are reported in Table 1. For both self and others, 5 factors were extracted. These factors corresponded to the dimensions we hypothesized (Nature, God, Other People, Self, and Chance). All items had moderate to high loadings on the appropriate dimension. One of the Chance items loaded less well (“basically chance is fair for me/people”; pattern/scale loading: .42/.59 and .33/.49 respectively). This is probably due to the phrasing of chance as an ‘actor’ in this context. Yet Confirmatory Factor Analyses revealed loadings of .61 and .51 respectively and alphas of the Chance dimension are high (α = .94 and α = .92 respectively).

**Table 1:** Pattern/scale loadings and communalities for the BJT5 scale for self and others (Step 1).

BJT5 for self	Pattern	Scale	Communality (after rotation)
**Nature (α = .95)**			
1. *I get what I am entitled to have due to the forces of nature.	0.83	0.84	0.71
2. *I feel that my efforts are noticed and rewarded by the forces of nature	0.93	0.91	0.84
3. I feel I earn the rewards and punishments I receive from the forces of nature	0.95	0.91	0.83
4. I feel that when I meet with fortune this is brought on me by the forces of nature	0.82	0.85	0.73
5. The forces of nature will ensure that I get what I deserve	0.89	0.89	0.80
6. I feel that the rewards and punishments that I get are fairly given by the forces of nature	0.93	0.91	0.82
7. *Basically the forces of nature are fair for me	0.69	0.71	0.55
Eigenvalue (percentage of variance explained)			7.35 (20.99)
**God (α = .99)**			
1. *I get what I am entitled to have from God.	0.95	0.95	0.91
2. *I feel that my efforts are noticed and rewarded by God	0.95	0.95	0.90
3. I feel I earn the rewards and punishments I receive from God	0.95	0.96	0.92
4. I feel that when I meet with fortune this is brought on me by God	0.95	0.95	0.90
5. God will ensure that I get what I deserve	0.96	0.96	0.93
6. I feel that the rewards and punishments that I get are fairly given by God	0.96	0.97	0.93
7. *Basically God is fair for me	0.93	0.92	0.85
Eigenvalue (percentage of variance explained)			9.65 (27.58)
**Other people (α = .95)**			
1. *I get what I am entitled to have from other people I encounter.	0.78	0.75	0.57
2. *I feel that my efforts are noticed and rewarded by other people I encounter	0.83	0.76	0.60
3. I feel I earn the rewards and punishments I receive from other people I encounter	0.83	0.83	0.69
4. I feel that when I meet with fortune this is brought on me by other people I encounter	0.78	0.78	0.62
5. Other people will ensure that I get what I deserve	0.71	0.76	0.60
6. I feel that the rewards and punishments that I get are fairly given by other people I encounter	0.85	0.87	0.77
7. *Basically other people are fair for me	0.76	0.76	0.59
Eigenvalue (percentage of variance explained)			2.13 (6.08)
**Self (α = .94)**			
1. *I get what I am entitled to have because of no one but myself.	0.87	0.81	0.68
2. *Whether my efforts are noticed and rewarded is determined by no one but myself	0.76	0.74	0.55
3. I feel I earn the rewards and punishments I get because of no one but myself	0.83	0.84	0.71
4. I feel that when I meet with fortune this is brought on me by no one but myself	0.88	0.89	0.79
5. I get what I deserve because of no one but myself.	0.86	0.87	0.78
6. I feel that the rewards and punishments that I get are fairly given because of no one but myself	0.88	0.88	0.79
7. *Basically I am treated fairly because of no one but myself	0.79	0.81	0.66
Eigenvalue (percentage of variance explained)			3.92 (11.21)
**Chance (α = .94)**			
1. *I get what I am entitled to have due to chance	0.84	0.84	0.71
2. *Whether my efforts are noticed and rewarded is determined by chance	0.91	0.85	0.74
3. I feel the rewards and punishments I receive are due to chance	0.91	0.89	0.80
4. I feel that when I meet with fortune this is brought on by chance	0.86	0.84	0.71
5. I get what I deserve due to chance	0.92	0.92	0.85
6. I feel that the rewards and punishments that I get are fairly given by chance	0.73	0.80	0.68
7. *Basically chance is fair for me	0.42	0.59	0.45
Eigenvalue (percentage of variance explained)			2.91 (8.33)
**BJT5 for other**			
**Nature (α = .95)**			
1. *People get what they are entitled to have due to the forces of nature	0.83	0.84	0.71
2. *I feel that people’s efforts are noticed and rewarded by the forces of nature	0.86	0.85	0.73
3. I feel people earn the rewards and punishments they receive from the forces of nature	0.93	0.90	0.82
4. I feel that when people meet with fortune this is brought on them by the forces of nature	0.89	0.89	0.79
5. The forces of nature will ensure that people get what they deserve	0.85	0.87	0.75
6. I feel that the rewards and punishments that people get are fairly given by the forces of nature	0.92	0.91	0.83
7. *Basically the forces of nature are fair for people	0.70	0.72	0.54
Eigenvalue (percentage of variance explained)			7.47 (21.33)
**God (α = .98)**			
1. *People get what they are entitled to have from God	0.95	0.95	0.89
2. *I feel that people’s efforts are noticed and rewarded by God	0.97	0.96	0.92
3. I feel people earn the rewards and punishments they receive from God	0.96	0.96	0.92
4. I feel that when people meet with fortune this is brought on them by God	0.92	0.94	0.87
5. God will ensure that people get what they deserve	0.96	0.96	0.92
6. I feel that the rewards and punishments that people get are fairly given by God	0.95	0.95	0.90
7. *Basically God is fair for people	0.91	0.91	0.83
Eigenvalue (percentage of variance explained)			9.98 (28.51)
**Other people (α = .95)**			
1. *People get what they are entitled to have from others they encounter	0.72	0.75	0.57
2. *I feel that people’s efforts are noticed and rewarded by other people they encounter	0.86	0.79	0.64
3. I feel people earn the rewards and punishments they receive from other people they encounter	0.83	0.80	0.65
4. I feel that when people meet with fortune this is brought on them by other people they encounter	0.83	0.80	0.65
5. Other people will ensure that people get what they deserve	0.75	0.79	0.63
6. I feel that the rewards and punishments that people get are fairly given by other people they encounter	0.79	0.82	0.69
7. *Basically other people are fair for people	0.63	0.69	0.51
Eigenvalue (percentage of variance explained)			1.84 (5.25)
**Self (α = .94)**			
1. *People get what they are entitled to have because of no one but themselves	0.80	0.78	0.61
2. *Whether people’s efforts are noticed and rewarded is determined by no one but themselves	0.80	0.78	0.63
3. I feel people earn the rewards and punishments they get because of no one but themselves	0.86	0.86	0.73
4. I feel that when people meet with fortune this is brought on them because of no one but themselves	0.87	0.86	0.75
5. People get what they deserve because of no one but themselves	0.88	0.87	0.75
6. I feel that the rewards and punishments that people get are fairly given because of no one but themselves.	0.87	0.89	0.80
7. *Basically people are treated fairly because of no one but themselves	0.78	0.82	0.67
Eigenvalue (percentage of variance explained)			3.84 (10.97)
**Chance (α = .92)**			
1. *People get what they are entitled to have by chance	0.80	0.80	0.64
2. *Whether people’s efforts are noticed and rewarded is determined by chance	0.85	0.84	0.71
3. I feel the rewards and punishments people receive are due to chance	0.92	0.90	0.81
4. I feel that when people meet with fortune this is brought on by chance	0.83	0.82	0.68
5. People get what they deserve due to chance	0.91	0.91	0.82
6. I feel that the rewards and punishments that people get are fairly given by chance	0.79	0.84	0.72
7. *Basically chance is fair for people	0.33	0.49	0.37
Eigenvalue (percentage of variance explained)			2.31 (6.60)

*Note*. *N* = 345

* = items of the reduced three item scale

This factor structure was replicated in Sample 3. Variances explained by each factor ranged from 3.21% (Chance-O) to 32.79% (God-O). As in Sample 1, all items loaded moderately to highly (.43 to .96) on the relevant dimensions. The four dimensions included in Sample 2 also replicated these dimensions. In sum, we conclude that these five dimensions can be replicated across a number of studies.

### Item Reduction

The high scale reliabilities of the subscales (with alphas>.91) in Sample 1 point to possible redundancy among items (e.g., [60], p. 121). Given the large number of items overall (7x6x2 = 84), we explored the possibility of item reduction without loss of validity or reliability. Thus we sought a compromise between high factor loadings for reliability and adequate heterogeneity of item content for validity [60]. Since all factor loadings were quite high, we chose to exclude items with higher factor loadings (across factors) first. We did so based on content criteria such as whether the items were very similar to another item and/or whether they could be considered less central to the BJW concept. We constructed scales consisting of 7, 6, 5, 4, 3, 2, and 1 item. We then computed the average R^2^ for each number of items with our validating scales. For this analysis we retained only those validating scales which correlated between .3 and .6 with the full 7-item scales. Since at this stage we are only concerned with assessing the reduction in variances explained for each item dropped from the scale, we do not report which scales were included for each factor (but see Step 3). Different scales met the correlation criterion for each factor (ranging from 1 to 5 scales). Where necessary, correlations were aggregated by Fisher-z transformation.

Normally, one would expect the R^2^ to go down as items are removed from a scale.

**Fig 1:**
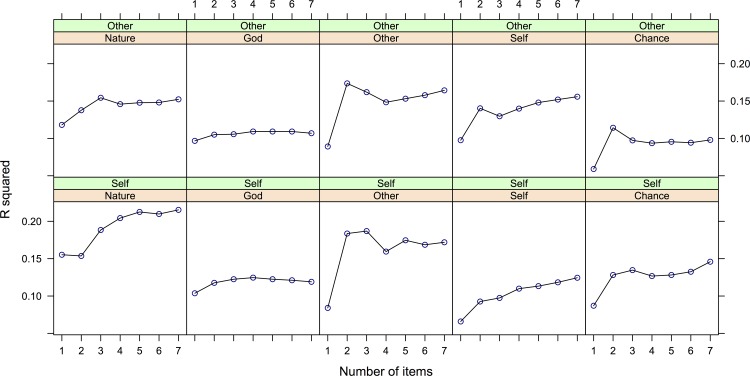
Average R2 for each number of BJT5 items with the validating scales (Step 1)

However, if the homogeneity of the underlying construct and item redundancy are high, R^2^ will not suffer from attrition as items are removed. Fig. 1 graphically displays the variances explained per scale. Based on these analyses and estimated reliabilities we concluded that a three-item solution (with items 1, 2, 7; see Table 1) was acceptable. The 3-item scales explained 95% of the variance explained by the 7-item scales, with reliabilities ranging from high to very high (.73 to .96, see Table 1).

## Step 2: Assessing Factor Structure

Theoretically, the literature has implicitly assumed that there might be multiple sources of justice, but that an operationalization of these multiple sources would be covered by the overarching concept of “the world” as a just place (e.g.,[2]). In assessing dimensionality of the BJW concept, one logical assumption would therefore be that the 5 separate factors are themselves subsumed by a general factor (the BJW). Moreover, at a methodological level, the BJT5 was designed by rewording versions of a previously validated scale. As a result, item stems are more or less the same from subscale to subscale, but the sources and targets of justice change. This necessarily means a lot of overlap in item content across subscales. Thus, for both theoretical and methodological reasons, subscales of the BJT5 could be highly correlated. Accordingly, in terms of dimensional structure, one would hypothesize there to be a general dimension of BJW. To test whether there is a general BJW concept that runs through our five dimensions of justice, one can specify a hierarchical model or a bifactor model, to account for the variance explained by the general factor (e.g., [61]).

The estimation of a bifactor model has one additional major advantage: it allows us to partition the common variance, estimating variance that is due to the general factor (e.g., a general dimension of BJW or an alternative individual difference) separately from group factors (e.g., the subscales asking about five sources of justice, see [62]). Omega H is a measure of reliability of the general factor: it estimates the variance attributable to the general factor, controlling for variance attributable to group factors. Omega S, conversely, estimates reliability of subscale scores while controlling for the variance attributable to the general factor (see [62], p. 137). This allows us to better evaluate the reliability of both the general factor and the subscales, because traditional measures of reliability (such as the alpha’s reported above) ignore the possibility that part of the reliability of the general factor is caused by the subscales, and vice versa. The omegas reported here tease the two apart and thus offer a statistical instrument to evaluate the utility of calculating either. Note that before testing the bifactor model described below we also attempted to establish model fit for the Lipkus self and other scales [6] yet it was not possible to determine a fitting model.

### Results and Discussion

We estimated a bifactor model in order to assess the dimensional structure of BJT5 [63, 64]. Therefore, we specified a general factor and 5 group factors, corresponding to the five dimensions of justice, all orthogonal with variances of 1, using Lavaan 0.5–17 [65]. Factor loadings are provided in S2 File. Because of the kurtosis of two subscales we report robust fit statistics with Satorra-Bentler adjustment. For the bifactor model for self the fit was χ^2^ (75) = 177.41, *p*<.001, CFI = .97, TLI = .95, RMSEA = .063 (.053–.072). The fit of the model for others was χ^2^ (75) = 225.62, *p*<.001, CFI = .94, TLI = .92, RMSEA = .076 (.067–.086). The fit is borderline if one strictly adheres to conventional benchmarks, but as noted by Reise et al. [62] such benchmarks have never been shown to be applicable for the evaluation of bifactor models based on polytomous items (see also [66]).

Factor loadings (S2 File) were healthy for all subscales (median = .70) and considerably lower for the general factor (median = .40). There was one exception to the pattern: God items loaded close to zero on the general factor (median = .05) and very highly on the group factor (median = .95). A closer inspection of the correlations between subscales shows why, but also identifies a potential empirical limitation of this bifactor model. Correlations among averaged items show Nature, Other People, Self, and Chance to be correlated in the .2 to .4 range, with average *r* = .32. But the correlations with the God factor are quite different: averaged across self and others, God correlates close to zero (−.09) with Other People, Self, and Chance. In contrast, God is correlated positively with Nature (.29 on average). Additionally, for items referring to others, God is correlated negatively with Chance (−.15). The variability of correlations with God are likely to cause residual covariance in an orthogonal model (i.e., the model cannot account for the fact that some factors are correlated and others not). Modification indices confirmed this.

Accordingly, we computed adjusted bifactor models in which cross-loadings were freed, allowing God to correlate with Nature (and Chance for others, too). The fit of the model for self was Satorra-Bentler χ^2^ (74) = 141.05, *p*<.001, CFI = .98, TLI = .97, RMSEA = .051 (.041–.061). The fit of the model for others was χ^2^ (73) = 163.40, *p*<.001, CFI = .96, TLI = .95, RMSEA = .060 (.049–.070). Now, the fit of both models was acceptable also according to conventional benchmarks. There were no additional covariances between factors that would have resulted in major improvements of model fit.

We calculated Omega H, indicating the ‘reliability’ for the general factor, and Omega S, indicating reliabilities for each of the separate dimensions, for each of these models (Table 2). In general, Omega S values are higher than Omega H. For the items referring to justice for oneself, Omega H for the general factor was estimated to be 44% (or 40% based on the adjusted model). Thus, a relatively small percentage of reliable variation is attributable to a single, general latent variable. Because of the low reliability one should interpret results of the general factor with care. Inspecting the values of Omega S for each subscale revealed that the reliability for Nature, Self, Other People, and Chance was considerably higher, values ranging from .55 to .67. Although not very high in absolute terms, these reliabilities meet the minimum threshold of .50 suggested by Reise et al. [62]. The God subscale was the one that had very high reliability (also because it shared almost no variance with the general factor).

**Table 2:** Estimated reliabilities for general factor and subscales, based on bifactor models for self and others in Step 2 (including bifactor models adjusted for covariation between God–Nature).

	Model for Self		Model for Others	
	Orthogonal	Adjusted	Orthogonal	Adjusted
**Omega H**				
General factor	0.44	0.40	0.54	0.52
**Omega S**				
Nature	0.58	0.57	0.48	0.50
God	0.97	0.94	0.95	0.96
Self	0.67	0.67	0.52	0.52
Chance	0.61	0.55	0.55	0.53
Other people	0.58	0.59	0.40	0.39

For the items referring to justice for others, Omega H was higher at 54% (or 52% for the adjusted model). This is just above the threshold value. A corollary of the higher reliability of the general factor is that subscales have less unique reliability. Accordingly, the only subscale that evidenced good reliability was God (.95/.96). Other subscales also had reliabilities around the threshold: Chance (.55/.53), Self (.52/.52), and Nature (.48/.50). Only the reliability for Other People was clearly below the threshold (.40/.39). This does not imply that the Other People subscale was lacking in reliability per se (see the good alpha above), but it *does* suggest that the reliability on this particular subscale is lower when the general factor is simultaneously taken into account (i.e., the general factor loads relatively highly on the items in this subscale). But this one reliability should not be interpreted independently of the broader pattern of results: Omega H is still on the low side relative to the “average” Omega S of the five subscales. The evidence suggests that while researchers interested in studying general factor scores can rely on them having at least some reliability, over all there is clear support that the subscales have adequate reliability (although not all of that is independent of the general factor).

From this, we conclude that there is clearly sufficient evidence to analyse and interpret the factor scores of the subscales. The reliability of subscales tends to be adequate, and that of the general factor tends to be lower (in particular for the self items). Of course the general factor still explains a proportion of variance: this could be explained by the considerable overlap in item content, but other explanations are also possible (such as response or social desirability biases). But importantly, even though the general factor can be estimated with some reliability, it still explains only a modest amount of variance on the subscales. This speaks against the idea that there would be an overarching belief in a just world that *strongly* shapes beliefs expressed in the subscales. 5. This inference is supported by correlations between predicted factor scores and established scales of belief in a just world, reported in S2 File. All things considered, it appears that there is clear support for our suggestion that it is beneficial to distinguish among sources of justice.

## Step 3: Convergent and Discriminant Validity

### Method

In order to further validate the BJT5, we assessed correlations with established BJW measures: Lipkus et al.’s [6] BJW scale for Self versus Others; Rubin and Peplau’s [31] BJW scale; Dalbert’s [67] Personal BJW scale. All scale responses varied from 1 (strongly disagree) to 7 (strongly agree). Note that part of the data (the Mturk participants of Sample 1) from the Lipkus self and other scale [6] were used to illustrate the three-mode factor analysis by means of Candecomp/Parafac in a recent paper on these novel statistical methods [68].

In addition, we administered measures that correlate highly with established BJW measures. Participants completed measures of fate control [69], life satisfaction [70], self-esteem [71], protestant ethic [72], religion (intrinsic religion; [73]), spirituality [69], conservatism, personality (extraversion, emotional stability; [74]), socio political control [50], and social dominance orientation (SDO; [75]). A measure of internal locus of control was ignored because of its low reliability.

### Results and Discussion

#### Relation with general BJW measures

Correlations were computed among the BJT5 and the general BJW scales (see Table 3, also for reliabilities, means, and standard deviations). Note that intercorrelations in Table 3 are based on scale averages of 3 items. We also calculated the correlations between factor scores of the full 2*5*7 item BJT5. For readers’ interest these are provided in S2 File; they do not deviate substantially from the correlations reported in Table 3.

**Table 3:** Alphas, means, standard deviations and correlations between the BJT5 and the just world scales (Step 3).

Scale	Alpha	M	SD	1.	2.	3.	4.	5.	6.	7.	8.	9.	10.	11.	12.	13.	14.
1.Nature-S	.86	3.18	1.56	1.00	.23**	.33**	.20**	.39**	.79**	.24**	.27**	.24**	.30**	.25**	.28**	.23**	.26**
2.God-S	.96	3.66	2.16	.23	-	−.02	−.11	−.17**	.26**	.86**	−.08	−.09**	−.18**	.06	.18**	.23**	.20**
3.Other people-S	.81	4.39	1.20	.33		-	.24	.22**	.23**	−.07	.59**	.25**	.17**	.54**	.28**	.33**	.53**
4.Self-S	.84	4.32	1.45	.20			-	.27**	.20**	−.09	.30**	.70**	.25**	.28**	.35**	.28**	.34**
5.Chance-S	.79	3.69	1.41	.39				-	.36**	−.13*	.34**	.28**	.74**	.03	.10	−.07	.03
6.Nature-O	.85	3.21	1.53	.79					-	.34**	.38**	.29**	.38**	.17**	.32**	.24**	.25**
7.God-O	.96	3.78	2.10	.24						-	−.03	−.05	−.15**	.03	.20**	.23**	.18**
8.Other people-O	.78	4.29	1.10	.27							-	.40**	.36**	.34**	.42**	.39**	.46**
9.Self-O	.83	4.26	1.35	.24								-	.31**	.30**	.45**	.30**	.40**
10.Chance-O	.73	3.69	1.34	.30									-	−.05	.09	−.08	.00
11. BJW for self (Lipkus et al.)	.89	4.72	1.08	.25										-	.57**	.55**	.76**
12. BJW for other (Lipkus et al.)	.91	4.01	1.16	.28											-	.60**	.69**
13. BJW (Rubin & Peplau)	.79	4.05	.68	.23												-	.72**
14. Personal BJW (Dalbert)	.93	4.36	1.06	.26													-

*Note*. *N* = 345

BJW = Belief in a Just World; S = self; O = other

*p <. 05

**p<.01

The BJW measures are highly correlated among each other (average *r* = .66), suggesting they broadly tap into the same construct. In addition, self and other judgments are highly correlated (BJT5 average *r* = .75; BJW_Lipkus_ *r* = .57). We can therefore form a general impression of the relationship between BJT5 and BJW by collapsing across self- and other as well as across BJW scales. We find correlations in the moderate range for two dimensions of BJT5: Nature (*r* = .25) and Self (*r* = .34). Correlations for God tend to be slightly lower (*r* = .17) and for Other People slightly higher (*r* = .42). Individual correlations (Table 3) are consistent with these conclusions. Interestingly, the Chance dimension did not correlate with any of the existent measures (average *r* = .00). Without undermining the plausibility of Chance as a determinant, it makes sense that it should be least related to existent measures, as it implicates a randomness to the world which could be seen as at odds with a belief in a world that is consistently just and fair [1]. Overall, these correlations indicate that the BJT5 has convergent validity in the sense that four of its dimensions are moderately correlated with the overarching BJW concept. Nevertheless, there is also considerable discriminant validity: the BJT5 and BJW scales tap into distinct constructs and the BJT5 dimensions are only moderately correlated among each other.

Prior work (e.g., [6, 21, 34]) reveals that people have a higher belief in a just world for themselves than for other people. In order to assess whether there were differences between the self versus other dimension, we ran a repeated-measures 2 recipients (self versus other) * 5 sources of justice (Nature, God, Other People, Self, Chance) ANOVA. Mauchly’s test indicated that the assumption of sphericity had been violated, χ2(9) = 42.96, p<.001, therefore degrees of freedom were corrected using Huyn-Feldt estimates of sphericity (ε = .96). There was no overall effect of recipient: across dimensions scores for self and other were almost identical, *F*(1, 344) = 0.05, *p* = .83, *η* ^*2*^<.001. Moreover, the 2*5 interaction effect was only marginally significant, *F*(3.83, 1318.04) = 2.27, *p* = .06, *η* ^*2*^ = .01. Given the near significance of this effect, we looked at simple main effects of all differences and found only one marginal difference for the dimension other people, with higher scores for ‘Other People-S’ (*M* = 4.39) than ‘Other People-O’ (*M* = 4.29); *F*(1, 344) = 3.47, *p* = .06, *η* ^*2*^ = .01. Other differences were not significant and descriptively small, *Fs*<2.64, *ps*>.10, *η* ^*2*^<.008.

Because prior work [6, 21, 67] has found that BJW self, but not other, is related to life satisfaction, we conducted regression analyses with the self and other measures included together separately for each dimension on life satisfaction. Results revealed that the Nature (β = .34, *t*(342) = 4.03, *p*<.001), God (β = .27, *t*(342) = 2.61, *p*<.01) and Other People (β = .47, *t*(342) = 7.83, *p*<.001) dimensions for self significantly predicted life satisfaction. The Self-self dimension marginally (β = .14; *t*(342) = 1.81, *p* = .06) and the Chance-self dimension did not predict (β = .05; *t*(342) = .63, ns) life satisfaction. In line with prior findings, when self and other were included as predictors, none of the dimensions for other predicted life satisfaction (βs <|.10|; ts.(342)<−1.18).

In sum, it seems that asking people to specify who makes the world just and fair for themselves and other people may reduce the bias towards seeing the world as more just and fair for oneself. At the same time, analyses for life satisfaction provide indications that the self versus other construct do have the differential effects on psychological adjustment found in prior work (e.g., [6, 32, 34]).

### Correlations with constructs related to the BJW

The belief in a just world has been found to correlate positively with life satisfaction, religiosity, political orientation (right wing, conservative), locus of control, cultural worldviews such as Protestant Ethic, and, although not consistently (e.g., [6]), with markers of personality.

Inspection of results revealed several distinct patterns between the BJT5 and these measures (see Table 4). All BJT5 dimensions except for Chance correlated moderately positively with life satisfaction, self-esteem, and protestant ethic. All BJT5 dimensions, except for the BJTSelf-Other People, showed moderate positive correlations with fate control. A somewhat different pattern emerged for the religion/political attitudes cluster. Here we found moderate to high positive correlations only between the BJT5 dimensions of God and Nature (with the exception of Nature and conservatism). The dimensions of Other People, Self, and Chance correlated moderately negative to not at all. Finally, the BJT5 showed very small, in some cases zero, correlations with measures of personality (extraversion, emotional stability), socio political control, and social dominance orientation.

**Table 4:** Correlations between the BJT5 and fate control, life satisfaction, self-esteem, protestant ethic, intrinsic religion, spirituality, conservatism, extraversion, emotional stability, socio political control and social dominance orientation (SDO) in Step 3.

BJW dimension	Fate control	Life satisfaction	Self-esteem	Protestant Ethic	Intrinsic religion	Spirituality	Conservatism	Extraversion	Emotional stability	Socio political control	SDO
BJT5-Self											
Nature	.43**	.26**	.11*	.22**	.14*	.19**	.03	.11*	-.02	.02	.1
God	.29**	.21**	.14**	.27**	.79**	.83**	.35**	.09	.09	.08	.02
Other people	.07	.45**	.31**	.18**	-.11*	-.08	-.06	.15**	.19**	.17**	.06
Self	.13*	.16**	.12*	.27**	-.21**	-.13*	-.01	.07	0	.08	.16**
Chance	.37**	−.01	−.02	−.02	−.20**	−.20**	−.19**	−.02	−.09	−.11*	.07
BJT5-Others											
Nature	.46**	.17**	.03	.23**	.14**	.23**	.07	.04	−.11*	−.03	.13*
God	.32**	.17**	.1	.28**	.71**	.78**	.33**	.04	.08	.04	.02
Other people	.21**	.24**	.17**	.17**	−.09	−.07	−.01	.04	.03	.08	.20**
Self	.17**	.13*	.13*	.33**	−.16**	−.08	−.01	.02	.03	.04	.19**
Chance	.31**	−.04	−.05	.0	−.19**	−.20**	−.1	−.05	−.1	−.13*	.04

*Note*. *N* = 344; SDO = Social Dominance Orientation

* *p*<.05

** *p*<.01

#### Conclusions

These results indicate that the BJT5 has discriminant validity: correlates of an overall BJW (e.g., religion, conservatism, life satisfaction, protestant ethic, personality, control) show distinctive relations with the separate dimensions of the BJT5. In addition, correlations between the BJT5 and these constructs were overall moderate to low, the only exception being the BJT5 God correlation with religion (*r* = .75). Results indicate that the strong relation between existent BJW scales and measures of religiosity found in prior research may have been driven by those people who feel God determines whether the world is just and fair. Given the God dimension is *one of* the dimensions with the highest explained variance it may have driven this effect.

Political ideology and especially conservatism, often correlate strongly with the BJW (e.g., [5, 76]. In line with a system legitimizing perspective, it has been suggested that politically conservative individuals and those who have a strong belief in a just world share an averseness to change and desire to maintain the status quo [5]. Interestingly, correlations between conservatism and the BJT5 dimensions only reveal positive correlations with the God dimension, again indicating that the correlation between conservatism and the BJW may have in part been driven by those people who feel the world is just and fair due to God.

Overall, results suggest that the BJT5 serves a distinct function compared to existent BJW correlates. We felt that the added value of the BJT5 should become even more evident when considering perceptions of and actions with regard to life events that may threaten the Belief in a Just world. We considered this in steps 4 and 5.

## Steps 4 and 5: Orientation Towards Life Events

In steps 4 and 5 we examined whether the BJT scale is differentially related to interpretations of life events and actions in response to these life events. For step 4, half of these events were in part designed to be potential threats to people’s BJW (e.g., a disaster with innocent people dying), the other half reflected positive events. In step 5 we looked at two of the negative events of step 4. Both steps were exploratory.

### Method Step 4

Participants of Sample 2 completed several scenarios that were designed to assess the utility of differentiating among BJT dimensions (3 participants did not complete the scenario measures). The scenarios are described in S3 File. They portrayed negative or positive life events either at macro level (i.e., occurring at a large scale to people in general) or at micro level (i.e., events that happen to you).

Scenarios systematically differed in the extent to which they might be directly attributable to different causal actors (Nature/God, Other People, and Self). The relation between these events and sources of justice as being ultimately responsible for these events was measured by asking participants to indicate per dimension (forces of Nature, God, Other People, Self) the extent to which each was responsible for the event described. Note that we expected sources of justice to predict perceptions of responsibility across scenarios, irrespective of potential direct attributions to causal actors. As noted above, we did not include Chance in this (chronologically) first study. Scale endpoints ranged from 1 (not at all) to 7 (very much).

One might question whether individuals would assign responsibility to a source of justice for an unjust event (given the need to maintain a view of the world as just). Yet we reasoned that by assigning responsibility to an underlying dimension, individuals are able to “encompass an incident of seeming injustice within the larger framework” in which there is an ultimate cause for incidents of injustice ([2], p. 164). We did predict that people should find it *easier* to assign responsibility for positive (just) rather than negative (unjust) events, as these events are congruent with the need to see the world as just. Thus, indicating agreement that the world is just due to other people means that an event portraying a *just* world as caused by other people more strongly matches people’s preconceived views of the sources of justice *and* the world as just. Consequently, we expected to find a stronger pattern of attributions to responsibility for the positive as opposed to the negative life events across causal actors.

### Results and Discussion

We were interested in exploring the possibility that perceptions of responsibility would be determined by endorsement of BJT dimensions such that *across* scenarios that varied in direct causality, high scores on one of the dimensions (e.g., BJT God) would induce higher perceptions of ultimate responsibility of the concurrent source (e.g., God) for the event described, regardless of who actually caused the event. We present the results separately for negative versus positive scenarios, but collapsed across type of scenario and self versus other (see Table 5). As expected, endorsement of a BJT dimension corresponded to perceptions of responsibility across scenarios for the dimensions Nature, God, Other People, and Self for the positive scenarios. Regarding the negative scenarios we found assignments of responsibility for Nature, God and, be it a small trend (*p* = .12), Other People. This stronger effect of the BJT5 for positive compared to negative scenarios is consistent with our expectation that it is easier to assign ultimate responsibility of one of the dimensions to positive rather than negative life events.

**Table 5:** Summary of linear regression analyses examining effects of the BJT with regard to self and other on perceptions of responsibility for life events (Step 4).

			BJT5Dimensions:							
			Nature		God		Other people		Self	
Attributions of responsibility	M	*SD*	*β*	*t-value*	*β*	*t-value*	*β*	*t-value*	*β*	*t-value*
*Negative events*:										
Nature	3.12	1.00	.32***	3.45	.04	.39	−.08	−.90	−.05	−.49
God	2.16	1.51	.13	1.63	.52***	6.27	−.07	−.81	.14	1.60
Other people	4.06	.75	−.12	−1.30	.11	1.13	.15	1.59	−.07	−.68
Self	3.11	.70	.09	.97	.04	.41	.07	.74	.04	.42
*Positive events*:										
Nature	3.62	1.32	.37***	4.07	−.02	−.23	−.08	−.84	−.07	−.74
God	3.38	2.18	.00	.04	.78***	13.48	−.07	−1.25	−.06	.32
Other people	3.90	.95	.11	1.20	.05	.51	.28***	3.06	−.01	−.05
Self	3.93	.96	.06	.69	.07	.73	.16	1.69	.24*	2.45

*Note*. *N* = 118 for negative events; *N* = 117 for positive events

*p<.05

**p<.01

***p<.001

Overall, these results provide evidence that the BJT dimensions have validity in that they are associated with differential perceptions of responsibility for both positive and, be it to a lesser extent, negative life events. Note that, when looking at regressions at the level of the scenarios, even negative events that had a strong element of own responsibility (i.e., riding through a red light and being hit by a car) were likely to be seen as caused by nature for those who endorsed the BJT Nature dimension (*β* = .33, *t*(117) = 3.63) or as caused by God for those who endorsed the BJT God dimension (*β* = .40, *t*(117) = 4.52). This means that higher order perceptions of causality (i.e., the BJT dimensions) can overrule direct indications of causality (e.g., *I* biked through a red light).

We next looked at whether the BJT is also associated with different types of action orientations in response to negative life events that have the potential to restore justice.

## Step 5: Action Tendencies in Response to Life Events

Researchers tend to stress that the belief in a just world may “discourage social activism” ([31], p.82) and empirical work looking at the relation between individual difference measures of BJW and action has supported this assumption (e.g., [24, 26]. For example, a study looking at student participation in demonstrations against student budget cuts revealed that even when students were able to redress their experiences of collective disadvantage by demonstrating, high BJW participants were less likely to demonstrate than low BJW participants [26]. Interestingly, in contrast to the individual difference literature, the BJW concept originally incorporated the idea that, whenever possible, individuals would be motivated to act out against injustice and re-establish justice (e.g., [1, 2]). Indeed experimental research revealed that inaction, such as blaming victims for their fate rather than helping victims by relieving their suffering, generally occurred when individuals were left with no means of addressing injustice (e.g., no available action; [1, 15, 77]).

Building on this original BJW idea, we reasoned that providing respondents with specific types of action that *befit* their predominant source of (in)justice establishes the necessary conditions for taking action. After all, one cannot appeal to “the world”. But if one believes that there is a God who will ensure that justice is done, one has a very clear course of action: to pray or make offerings. Similarly, if other people are deemed responsible for justice in the world, one can appeal to them or their institutions. In studies looking at the BJW, these multiple sources of injustice are confounded into a notion of “the world” that is suited to project any type of belief upon. This may explain why BJW does not explain well what actions individuals take upon the injustice they encounter. In line with the BJW literature which often focuses on actions directed at victims of injustice (but see [77, 78]), this study considered actions people would take regarding victims of injustice.

Concretely, we expect that when a course of action is congruent with individuals’ source beliefs, they will act out against injustice. For example, individuals who believe that other people are the source of justice should support aid organizations that help victims. In addition, based on the personal control literature [10, 45, 55], we reasoned that certain sources (God, Nature, Other People) should be more likely to induce action-oriented responses than others (Chance).

We chose two scenarios of which we knew that they induce perceptions of responsibility across all BJT5 dimensions based on the analyses from step 4. Although this should not affect the ability of the BJT5 to predict action responses across both scenarios, we chose one scenario in which the causality of action was more external and on in which it was more internal: the scenario in which parts of the world are destroyed by storms and floods (i.e., nature; external) and the scenario in which one puts a firework manual on YouTube which costs people’s lives (i.e., self; internal). These extremes are interesting for studying action responses: They could elicit a wide range of responses, varying from inaction in response to an externally caused event (i.e., a natural catastrophe) that may seem out of control, to many types of action for an internally-caused event. Yet we expected that by offering individuals actions that were congruent with the BJT5 dimension they endorsed, we would find action befitting the source of justice both in response to the externally and internally caused scenario.

### Method

Participants of Sample 1 were presented with an externally (natural catastrophe) and an internally (i.e., other people’s deaths due to own firework manual) caused event, asked to think about the event described, and to consider what they would do in response to this event. In the latter case participants indicated agreement with the following action responses for the external and internal events respectively: a 2 item measure of religiously oriented action (“I would give money to a religious organization that is helping the victims; I would pray to God to help the victims”; α_e_ = .73/α_i_ = .73), a 3 item measure of other people/human institution oriented action (e.g., “I would support a group of people I know who are helping the victims”; “I would support organizations such as Red Cross, United Way, Peace Corps and Unicef”; α = .66_e_/α_i_ = .69), personal action (“I would try to go to the affected regions to help”), and a 3 item measure of personal inaction (e.g., “I would do nothing, all will turn out well in the long run”; I would do nothing in first instance, these situations generally resolve themselves”; α_e_ = .90/α_i_ = .93).

### Results and Discussion

We conducted regression analyses across scenarios on the separate action/inaction scales, including a combined measure of the BJW Self and Other scale [6] at step one and a combined measure of the BJT5 Self and Other scales at step 2 of the regression analyses (see Table 6). Note that separate analyses for these two scenarios yielded the same pattern of results although the BJT5 Nature dimension was somewhat more related to inaction in the nature than in the self scenario (β = .16, *t*(343) = 2.45, p<.05 and β = .07, *t*(343) = 1.05, n.s., respectively; see S1 Table for analyses per scenario).

**Table 6:** Summary of hierarchical regression analyses examining effects of the combined BJW self and other scale (Lipkus et al., 1996) and the BJT5 on action type (Step 5).

Predictor	Action type	M(SD)	Model 1		Model 2	
			*β*	*t-value*	*β*	*t-value*
	*God*	3.86(1.96)				
BJW (Lipkus)			.10^†^	1.81	.00	.04
Nature			-		−.11*	−2.64
**God**			-		**.83** ***	**22.35**
Other people			-		.03	.60
Self			-		−.01	−.19
Chance			-		.02	.45
R^2^			.01		.63	
ΔR^2^					.64***	
	*Other people*	4.72(1.18)				
BJW (Lipkus)			.10	1.88	−.06	−.96
Nature			-		−.01	−.21
God			-		.29***	4.89
**Other people**			-		**.24** ***	**3.72**
Self			-		.02	.29
Chance			-		.00	.03
R^2^			.01		.10	
ΔR^2^					.10***	
	*Self*	4.24(1.59)				
BJW (Lipkus)			.05	1.00	−.07	−.99
Nature			-		.10	1.58
God			-		.27***	4.56
Other people			-		.03	.40
**Self**			-		**.08**	**1.35**
Chance			-		.01	.20
R^2^			.00		.10	
ΔR^2^					.10***	
	*No action*	2.63(1.43)				
BJW (Lipkus)			.13**	2.47	.09	1.42
Nature			-		.11^†^	1.75
God			-		−.07	−1.12
Other people			-		−.10	−1.54
**Self**			-		**.12** *	**2.03**
Chance			-		.19**	2.98
R^2^			.02		.09	
ΔR^2^					.11***	

*Note*. *N* = 345

†p<.1

* *p*<.05

** *p*<.01

***p<.001

The present analyses allowed us to assess whether the BJT5 dimensions predict action, and to what extent they are able to do so over and above a general BJW measure. To aid interpretation of the effects, the predicted associations are printed in bold.

The first thing to note in Table 6 is that, whereas BJW explains nearly no or very small amounts of variance in step 1 of these analyses, the inclusion of the BJT5 in step 2 leads to a major increase in variance explained for every single analysis. The second thing to note is that three out of the four predicted effects for BJT5 congruent action were statistically significant (75%). However, there were also quite a few unexpected effects: 4 out of 20 (20%).

Interestingly, although the BJT God dimension clearly predicted God-related actions most strongly, it also predicted actions towards other people and self, but was unrelated to inaction. Looking at the Self and Other People items this makes sense as they do not preclude God-related action, self-related action could be seen as missionary activities (“I would try to go to the affected regions to help”) and other people related action could relate to other religious persons within one’s community ("I would support a group of people I know”).

Furthermore, with respect to God-directed action, there was a substantial effect of BJT God, combined with a negative effect of the BJT Nature dimension revealing that those who believed these effects to be caused by nature showed slightly lower inclinations to take God-related action. Although we had not anticipated this effect, it makes sense.

As expected, the Other People dimension predicted other-people directed actions: the more other people were seen to be able to ensure just outcomes, the more they were targeted to take action.

Prior research has often shown that general BJW measures are related to inaction. The present research replicates this finding. But in the second step of this analysis, the small effect of BJW is entirely mediated by stronger effects of the Self and Chance dimensions of the BJT5. Again, we did not anticipate the effect of Chance on inaction, but given the nature of the items used this effect makes sense: the nature of the inaction items suggests that indifference (not-my-problem) could have prompted people to endorse these particular inaction items. The lack of action for the Self dimension can be understood when one considers we are assessing whether a participant felt he/she should take action to help a victim. Seeing the self as responsible for justice implies that it is the victim’s (and not a third person’s) responsibility to act. Although not the focus of the present studies, this reasoning has interesting implications in relation to victim derogation, blaming the victim for his/her injustice, which is widely studied in relation to the BJW and shown to correlate highly, and positively with it (see [1, 4]): It is likely that the BJT5-Self with its strong focus on personal responsibility, would be most strongly associated with victim derogation. This is an interesting avenue for future research.

In sum, although there were some unexpected findings we also confirmed the predictions: BJT5 differentially predicts behavioral outcomes over and above a general BJW measure. Results confirm that in order to predict specific action intentions, it is useful to differentiate between dimensions of BJT. The next study sought to more carefully manipulate the motives behind inaction, in order to tease BJT5 components apart.

## Step 6: Societal Attitudes towards Criminal Behavior

In step 6 we tested two ideas. The first was to examine how BJT5 relates to societal attitudes, specifically perceptions of criminal behavior. The literature suggests that BJW affects people’s societal attitudes such as responses to low status groups (e.g., [21, 32]). This approach moves away from studying direct experiences of victims of injustice, to more broadly assessing how the BJW affects societal attitudes such as those towards stigmatized persons. In the present step we aimed to illustrate that the specific nature of these attitudes is predicted by BJT5.

Secondly, we wanted to extend the finding that BJT5 predicts people’s action intentions (Step 5) by studying whether BJT5 also predicts specific motives for *inaction*. Part of the reason for this focus is that in most situations inaction appears to be the dominant response: it is actually very hard to mobilize people to undertake any action at all (e.g., Wright et al., 1990). Thus, although the majority of the literature focuses on the question of what motivates action, or how to overcome inaction, it is quite rare to examine inaction as a phenomenon in its own right. And yet, if inaction is indeed the dominant response in many situations, it would appear to be the more important phenomenon to understand. For this reason, in our own recent research, we have begun to explore multiple motives that may underpin collective inaction ([26, 79]). Up to this point the literature has identified several motives for action, but we believe that inaction may also be motivated by quite different reasons. The present research focuses on the topic of criminal behavior because it can have such markedly different origins. The specific motives for inaction in the present study were operationalized by reference to these possible origins (e.g., it is up to criminals to help themselves). As a first step to probing the relation between BJT5 and inaction, the present research examines a basic hypothesis: that the BJT5 dimensions would relate to these specific reasons for inaction. For example, someone with a higher score on the BJT5-Nature should be more likely to endorse nature related reasons for inaction (e.g., cannot help due to genetic predisposition for criminal behavior) than other reasons (e.g., it is up to criminals to help themselves).

### Method

Participants of Sample 4 filled in a combined self and other BJT5 scale and were asked to think about criminal behavior via the following description “If you look at the lives of different people you see that some of them lose control of their lives and start engaging in criminal behavior”. Then, *perceived responsibility for crime* was measured by asking participants to indicate why they thought people engage in criminal behavior and consisted of a two item measure of nature-related responsibility (due to genetic predisposition; because they are born this way and cannot help themselves; *r* = .61), a two item measure of God-related responsibility (“due to God’s will”; “because they have left the path God designed for them”; *r* = .77), a two item measure of other people-related responsibility (“due to lack of help from institutions that should prevent criminal behavior; e.g., schools that do not help problem children, justice system that punishes rather than treats criminals”; “due to bad parental upbringing”; *r* = .35-due to low inter item correlations we considered these items separately), a two item measure of self-related responsibility (“due to themselves”; “because they chose to do so”; *r* = .73), and a one item measure of chance (“by bad luck: being in the wrong place at the wrong time”).

The questionnaire then asked a few questions about different kinds of action (e.g., we should punish them, we should help them mend their ways). But in this study we did not break down the different actions as systematically as in the previous step and we do not analyze these items in this paper: the main focus was on inaction and the main reason for asking these questions was to justify asking several questions about different motives for not doing anything. Motives for *inaction* were then assessed by asking participants to indicate why they would choose *not* to help these people (see also [79]). Motives for inaction were nature-related (“we cannot really help these people much, they are genetically predisposed to engage in such behavior”), God-related (“there is little we can do to help these people as what happens to them is God’s will”), other people-related (“with society and the justice system as it is, there is little we can do to help”), self-related (“we cannot really help these people much, it is up to them to help themselves”), and chance-related (“we cannot really help these people, that’s just one of those bad turns life took for them”; “we cannot really do much but hope that the next time they don’t engage in criminal behavior”; “no action is possible, some will and some won’t engage in criminal behavior in the future”; α = .83).

### Results and Discussion

We expected that the responsibility for crime should map onto BJT5 dimensions, so that each BJT5 dimension predicts concurrent attributions of responsibility. For example, BJT5-self should uniquely predict seeing criminal behavior as the choice of the criminal him/herself. Multiple regression analyses are presented in Table 7. For the ease of readers we printed those regression coefficients which we expected to be significant in bold. Looking at the top half of the Table, we see that 5 out of 6 predicted effects were statistically significant (83%). By contrast, of the 30 other regression coefficients only two were statistically significant (7%). We thus found strong support for our predictions.

**Table 7:** Summary of regression analyses examining effects of BJT5 self and other on attitudes towards criminal behavior (responsibility and inaction).

			BJT5 dimensions:									
			Nature		God		Other people		Self		Chance	
	*M*	*SD*	*β*	*t-value*	*β*	*t-value*	*β*	*t-value*	*β*	*t-value*	*β*	*t-value*
***Attributions of responsibility*:**												
Nature	3.04	1.32	**.23** *	**2.20**	.01	.01	.15	1.57	−.10	−1.06	.34**	3.58
God	1.54	1.06	.03	.30	**.35** **	**2.99**	−.02	−.22	.02	.17	.02	.19
Other people:												
*Upbringing*	5.53	1.08	.12	1.08	−.13	−1.04	**.20** *	**1.99**	−.04	−.40	.03	.26
*Justice system*	5.29	1.52	−.06	−.57	.03	.21	**.16**	**1.61**	−.06	−.67	.29*	2.87
Self	4.70	1.40	.02	.22	.06	.54	−.08	−.83	**.34** ***	**3.76**	.05	.65
Chance	4.68	1.36	−.05	−.46	−.03	−.21	−.01	−.11	−.03	−.30	**.28** **	**2.75**
*Inaction*:												
Nature-related	1.99	1.10	**.32** **	**3.01**	−.20	−1.83	.17	1.84	.09	.96	.19*	2.03
God-related	1.36	.95	.05	.49	**.35** **	**3.04**	−.05	−.47	.09	.99	.16	1.63
Other people-related	3.09	1.31	.14	1.20	−.05	−.37	**−.11**	**−1.03**	.14	1.38	.03	.28
Self-related	2.75	1.56	−.07	−.63	.02	.16	.05	.47	**.25** *	**2.52**	.08	.79
Chance-related	1.96	1.06	.04	.39	.16	1.36	.12	1.22	.26*	2.72	**.21** *	**2.15**

*Note*. *N = 106*

*p<.05

**p<.01

***p<.001

Additionally, we also found that BJT5 dimensions could predict specific motives for inaction. For example, nature related inaction (cannot help these people due to genetic predisposition) was uniquely predicted by the BJT5-Nature and by no other dimensions. Looking at the bottom half of Table 6, we see that 4 out of 5 predicted effects were statistically significant (80%). By contrast, only one out of 20 other regression coefficients was statistically significant (5%). Again, there was strong support for predictions on all dimensions except for the item referring to other-people related inaction (no action possible with the nature of the justice system). However, since this item did not relate to any of the other BJT5 dimensions either, the operationalization of this item may have been suboptimal. Notwithstanding this one null effect, overall step 6 reveals strong support for the hypotheses.

Two points should be noted. First, in some cases the formulation of responsibility or motive for inaction items contain elements of causal attribution (e.g., “there is little we can do to help these people as what happens to them is God’s will”) that conceptually overlap with the BJT5 sources of justice (e.g., basically God is fair to me). For these dimensions the results should be interpreted with some caution due to the risk that these variables may be somewhat confounded. But on other dimensions (such as Nature) this overlap is much less obvious. For example., BJT items such as “basically the forces of Nature are fair for people” and the inaction item “we cannot really help these people much, they are genetically predisposed to engage in such behavior” are clearly unconfounded.

The second point is that the way we assessed motives for inaction does not preclude the possibility that people might still be motivated to engage in action to punish or help criminals. After all, participants only indicated the extent to which they felt that criminals could not be helped for various reasons. The fact that these motives for inaction mapped onto the five dimensions of the BJT5 is therefore not inconsistent with the findings of Step 5, that different sources of justice may predict action motivation, too. In sum, BJT5 helps us understand perceptions of responsibility for negative life events, preferences for particular courses of action (Step 5), as well as potential reasons for inaction (Step 6).

## General Discussion

The belief in a just world is a fundamental force in determining individuals’ perceptions of and responses to unjust life events. In this paper we raised the question of what or who is responsible for the world being just (or not). Our work demonstrates that at least five sources of justice (Nature, God, Other People, Self, Chance) can be distinguished that map onto five relatively independent dimensions. These five sources are held responsible for distributing justice and shape both people’s perceptions of and (in)action tendencies in response to injustice.

From a theoretical perspective, the present work is interesting as it provides better insight into the BJW concept, more specifically whether it is an *overarching sense of justice* versus an entity filled with people’s personal beliefs about the causal elements that ultimately define the world. The question of uni- versus multidimensionality is one that has been raised in previous work [5]. For example, the distinction of a BJW for self or for others (the question of justice for whom) is differentially related to important behavioral outcomes [6, 32, 34]. Across a number of diverse samples, and using a variety of methods and analyses, the current work provides evidence for multi-dimensionality: analyses clearly support that it is useful to distinguish five dimensions. These are differentially related to important correlates (e.g. life, satisfaction, societal attitudes). Evidence for an underlying general factor overall is marginal. To the extent that there is a general factor, it explains only a modest amount of variance and may not be estimable with sufficient reliability. Our findings thus suggest that researchers interested in studying the general factor should do so with some prudence. Distinguishing the BJW into five sources of justice may offer somewhat more scope for enhancing our understanding of people’s responses to injustice.

As such, the present findings also speak to recent discussions regarding the relation between different BJW operationalizations and ‘the justice motive’. The justice motive “has as its goal the ability to believe or assume that the world is just” and is seen to be universal, such that everyone has the desire to maintain and, in the face of threat, to retain it [80]. Because the implication of such a motive is that virtually everyone should have the ultimate *need* to believe in a just world, it has been questioned whether a scale looking at dispositional differences in whether people believe the world to be just may fully correspond to the underlying idea of such a motive [80, 81]. It has been proposed that, in line with the experimental just world literature, and an underlying justice motive, a focus on situational pressures as well as individual *differences in strategies* used to achieve or retain one’s sense of justice may more directly relate to our conceptualization of the justice motive[80, 81]. Underlying this reasoning, we believe, is the idea that how people conceptualize justice and make sense of injustice may take on very different forms, as is also illustrated by the BJT5. We have distinguished five potential sources of justice, but ultimately there may be more ways in which people fill in their conceptualization of justice (e.g., fate, karma). Some of these may relate more strongly to a desire for ultimate justice or be better able to fulfill the need for justice, such as the BJT5 God dimension, than others, such as the BJT5 Chance dimension.

The present work has important implications regarding the just treatment of the self versus of other people. In contrast to prior work comparing this distinction (e.g. [6]), we did not find that people felt the world was more just for themselves than for other people across the BJT5 Self versus Other dimensions. Indeed, the BJT5 Self versus Other dimensions were quite highly correlated (.59–.86), whereas the BJW for self and other correlate somewhat less at .57. It seems plausible that being prompted to think about the sources of one’s own deservingness (e.g., Chance, Nature, God) reduces the bias to see the world as more just for oneself than others: There may be less reason to believe that one should personally be blessed by favors of these concrete agents than other people should. Notwithstanding the very high correlations between self and other perceptions, differential psychological consequences of the Self-Other distinction were evident. There was a stronger relation between BJW Self than Other on psychological adjustment (e.g., life satisfaction). Conversely, some of the BJW Other (Other People and Self as well as, marginally, Nature) but not the BJT Self dimensions were related to societal attitudes (e.g., SDO). Our results seem to indicate that people do not necessarily have to see the world as more just for themselves than for other people to experience the ‘benefits’ of psychological adjustment.

Interestingly, the BJT5 sources of justice are able to predict the nature of perceptions of responsibility for important life outcomes and criminal behavior. For example, someone scoring high on the self as a source of justice is more likely to see criminal behavior as a personal choice. The strength of the BJT5 to predict perceptions of responsibility is also illustrated by its predictive validity for a wide range of negative life events that varied in the ‘direct’ causality attributions one could make for these events (e.g., a natural catastrophe versus a self-caused bike accident). Here, even negative events that had a strong element of own responsibility (i.e., riding through a red light and being hit by a car) were likely to be seen as caused by nature for those who endorsed the BJT Nature dimension. From an applied perspective, knowledge of whom people are likely to consider responsible for negative outcomes, such as criminal behavior, can have far reaching implications. Indeed, as our research shows, seeing genes or the criminal him/herself as responsible for behavior has implications for the type of treatment endorsed against criminals. This may be quite consequential: consider jury decision making when perceptions of responsibility involve making judgments regarding potential courses of action against a criminal up for trial. The example of a criminal who avoided the death penalty because his defense team argued he possessed a genetic mutation (shared by 33% of the population) which could diminish culpability, illustrates people’s vulnerability to different types of underlying causal attributions [82, 83]. The present work indicates that it is not only the possible attributions people are ‘provided’ with, but also their individually held perceptions regarding sources of justice that may have a strong impact on decision making.

The present approach also has interesting theoretical implications concerning the relation between BJW and responses to injustice. Although it is generally assumed that people respond to threats to their just world belief with a need to restore justice, this ‘restoration’ is not very ‘proactive’: people often blame victims of injustice for their fate rather than taking action to address their injustice (e.g., [1]). When faced with *personal* injustice, which has received less empirical attention, those with a high BJW are *less* likely to take collective action against it [24–26]. Thus, there is a general assumption both regarding injustice to others and to the self that the BJW is associated with inaction.

In line with the above, we found that the BJW predicted inaction but failed to predict action. Yet our findings for the BJT5 support a more differentiated perspective. In the present work we related the BJT5 to action tendencies in response to different life events in which other persons experience injustice. Although ideally, we would have liked to measure ‘real’ action, the present approach allowed us to customize and consider responses to major life events designed to correspond to the different BJT5 levels (e.g., Nature, Self). The present research demonstrates that the BJT5 dimensions may be related to both specific types of social action as well as specific types of inaction.

Firstly, BJT5 is better able to predict social action when there is a match between the type of action available and the perceived source of justice. For instance, belief in a just treatment by Other People was only related to other-people type action and not to other types of action. Further, BJT5 dimensions predicted specific motives for not taking action. Secondly, *whether* people act out against injustice, depends on *whom* they believe ensures the world is just. Those who have a strong belief in God as determining the world as just are likely to engage in a variety of actions in response to unjust life events—including helping victims of injustice directly. By contrast, the dimensions of Self and Chance are related to inaction. These results would seem to indicate that the failure of the BJW to predict action might simply be due to the fact that action directed at “the world” is too abstract and unlikely to be efficacious. The present work underlines the pragmatic utility and validity of the BJT5, highlighting the advantages of specifying the source of BJW.

Our research demonstrates that it is not the case that the more powerful ultimate causes of just treatment (e.g. Nature, God) would subjectively disempower the individual to engage in action. If God, or some supernatural forces, determines justice, human intervention could be seen to be superfluous. This would make sense given that uncontrollable, unjust events, such as one’s child being diagnosed with cancer, conversely increase attributions of causality to God [46, 84, 85]. It would have seemed plausible had God been invoked particularly when people feel personally powerless against negative life events. But the results actually suggest the opposite: the dimensions God and Nature show the strongest relation to action, despite the fact that individuals clearly perceived God and Nature as responsible for these actions (see Step 4). Similarly, work considering the relation between religion and prosocial behavior often,but not always, reveals that people who have a stronger belief in God are more likely to engage in prosocial behavior (see [86] for a review). From this perspective it appears that the more powerful and controlling individuals see causal forces to be, the more they feel energized and empowered to take action themselves. Interestingly, when the Self or Chance are considered responsible for justice, feelings of powerlessness, or at least lethargy, in the face of possible action against injustice appear to be most evident—even when personal action to address injustice would seem viable (e.g., personally caused injustice and self-action to address this outcome).

We conclude that, in providing an answer to Lerner’s question of how people cope with salient injustice in this world, considering the causal dimensions underlying the BJW is essential. Our work provides strong indications that the BJW, rather than being an overarching sense of justice, may consist of individual belief systems regarding ‘who’ distributes deservingness. It asserts that these individual belief systems matter; they are differentially related to important correlates of the BJW as well as to perceptions of and responses to negative life events. So in responding to Lerner’s initial question of how people cope with and respond to salient injustices in this world, we would counter that the question “who distributes injustice” is an essential part of the answer.

## Supporting Information

S1 Dataset Dataset for sample 1. (SAV)

S2 Dataset Dataset for sample 2. (SAV)

S3 Dataset Dataset for sample 3. (SAV)

S4 Dataset Dataset for sample 4. (SAV)

S1 File Exploratory statistical analyses of Belief in a Just World data. (PDF)

S2 File Supplementary analyses for Step 2—assessing uni- or multi-dimensionality of the BJT5 via bifactor analyses. (PDF)

S3 File Scenarios in Sample 2. (PDF)

S1 Table Summary of hierarchical regression analyses examining effects of action type in the Nature versus Self scenarios (Step 5). (PDF)
