# Supplementary material for: Belief in a Just What? Demystifying Just World Beliefs by Distinguishing Sources of Justice
Source: PLoS One. 2015 Mar 24;10(3):e0120145. doi: 10.1371/journal.pone.0120145 (PMC4372594; doi:10.1371/journal.pone.0120145)
Supplement: S1 File — (PDF) [file pone.0120145.s005.pdf]

Exploratory statistical analyses of Belief in a Just World data - part of which support Step 1 (i.e., combination of online and lab data)

Alwin Stegeman

Data received on January 28, 2013.

7 items with scores 1,2,3,4,5,6,7
6 actors: Nature, God, Human Institutions, Other People, Yourself, Chance
2 perspectives: for Other People, for Yourself

study 1: USlab data (students, N=109 after deleting subjects with missing data)
study 2: Mturk data (online volunteers, N=236 after deleting subjects with missing data)

Descriptive Statistics: means, standard deviations, frequencies

Table: means and standard deviations per actor, perspective, and study, for all items together.

	Others (study 1)	Yourself (study 1)	Others (study 2)	Yourself (study 2)
**Nature**	3.20	3.23	3.06	2.92
	(1.71)	(1.72)	(1.76)	(1.81)
**God**	3.17	3.21	3.92	3.76
	(2.04)	(2.08)	(2.21)	(2.28)
**Institutions**	4.12	4.20	4.09	3.89
	(1.35)	(1.36)	(1.42)	(1.51)
**Other People**	4.36	4.48	4.21	4.06
	(1.35)	(1.31)	(1.37)	(1.53)
**Yourself**	4.56	4.62	4.22	4.29
	(1.44)	(1.50)	(1.58)	(1.68)
**Chance**	3.74	3.62	3.68	3.63
	(1.53)	(1.53)	(1.70)	(1.76)

Remarks:

- In study 1 the means are generally larger for Yourself than for Others, while the converse is true in study 2 (due to the self-confidence of youth?).
- Standard deviations are larger for study 2 than for study 1 (80 of 84 items), probably due to less homogenous sample (larger age range, not all with academic background).
- Standard deviations are larger for Yourself than for Others for study 2 (39 of 42 items) but not for study 1 (24 of 42 items).
- Standard deviations are largest for God in both perspectives and studies, perhaps due to differences in ideas about ‘God’.

study 1	item 1	item 2	item 3	item 4	item 5	item 6	item 7

**answer 1**	142	159	164	155	193	183	158
**answer 2**	134	142	133	120	166	157	139
**answer 3**	206	197	192	206	203	207	183
**answer 4**	297	262	273	251	261	265	300
**answer 5**	317	312	316	326	281	306	267
**answer 6**	151	180	179	197	145	141	175
**answer 7**	61	56	51	53	59	49	86
**study 2**	**item 1**	**item 2**	**item 3**	**item 4**	**item 5**	**item 6**	**item 7**
**answer 1**	388	408	400	401	486	471	419
**answer 2**	317	335	351	345	387	384	281
**answer 3**	391	377	374	360	372	393	374
**answer 4**	635	550	559	574	576	591	681
**answer 5**	565	589	595	614	504	523	512
**answer 6**	364	386	379	367	319	309	365
**answer 7**	172	187	174	171	188	161	200

Table: Frequencies of answers for each item, for all actors and perspectives together.

Remark:

- Although frequencies of answer 7 are considerably lower than the rest, they are not low enough to consider merging answering categories 6 and 7.

Preliminary analysis of item correlations

**study 1**	**item 1**	**item 2**	**item 3**	**item 4**	**item 5**	**item 6**	**item 7**
**item 1**	1	0.74	0.76	0.74	0.70	0.70	0.57
**item 2**	0.79	1	0.80	0.75	0.76	0.77	0.60
**item 3**	0.74	0.76	1	0.77	0.74	0.79	0.60
**item 4**	0.76	0.73	0.78	1	0.75	0.75	0.61
**item 5**	0.72	0.70	0.77	0.74	1	0.83	0.70
**item 6**	0.75	0.75	0.83	0.78	0.85	1	0.70
**item 7**	0.66	0.65	0.69	0.67	0.71	0.78	1
**study 2**	**item 1**	**item 2**	**item 3**	**item 4**	**item 5**	**item 6**	**item 7**
**item 1**	1	0.77	0.78	0.75	0.75	0.74	0.64
**item 2**	0.76	1	0.82	0.76	0.75	0.75	0.63
**item 3**	0.80	0.81	1	0.79	0.79	0.80	0.65
**item 4**	0.78	0.75	0.80	1	0.78	0.79	0.62
**item 5**	0.78	0.76	0.79	0.77	1	0.83	0.68
**item 6**	0.75	0.77	0.81	0.78	0.82	1	0.71
**item 7**	0.67	0.71	0.66	0.67	0.68	0.72	1

Table: Item correlations for all actors together, and perspectives for Others (above the diagonal) and for Yourself (below the diagonal).

Remark:

- All items are highly correlated (with item 7 slightly lower) in both studies.

study 1	Nature	God	Institutions	Other People	Yourself	Chance
Nature	1	0.36	0.23	0.24	0.20	0.29
God	0.20	1	0.01	-0.09	-0.00	-0.12
Institutions	0.27	0.02	1	0.65	0.26	0.16
Other People	0.14	-0.16	0.66	1	0.29	0.31
Yourself	0.09	-0.06	0.14	0.20	1	0.15
Chance	0.36	-0.17	0.23	0.30	0.12	1
**study 2**	**Nature**	**God**	**Institutions**	**Other People**	**Yourself**	**Chance**
Nature	1	0.34	0.31	0.29	0.19	0.33
God	0.26	1	0.09	0.02	-0.05	-0.10
Institutions	0.29	0.07	1	0.71	0.35	0.22
Other People	0.34	0.03	0.73	1	0.38	0.25
Yourself	0.18	-0.15	0.25	0.23	1	0.25
Chance	0.38	-0.13	0.20	0.22	0.25	1

Table: Correlations among actors for all items together, and perspectives for Others (above the diagonal) and for Yourself (below the diagonal).

Remark:

- In both studies, the actor correlations are fairly low except between Human Institutions and Other People.

T-tests to compare means for the two perspectives

We do a paired sample t-test for each item and actor separately for the two studies. Hence, we obtain 42 t-values for each study.

study 1	item 1	item 2	item 3	item 4	item 5	item 6	item 7
Nature	0.48	0.28	0.19	1.39	0.39	-1.44	**-2.91**
God	-0.07	0.19	-0.47	0.21	-0.44	-0.59	-1.11
Institutions	-0.85	-0.69	0.14	0.77	1.21	-0.41	**-5.19**
Other People	-1.43	**-2.93**	0.26	0.00	**2.53**	0.17	**-5.46**
Yourself	1.69	1.07	0.14	1.38	**-2.09**	**-2.08**	**-3.27**
Chance	1.19	1.16	**2.71**	**2.71**	1.21	0.53	**-1.97**
**study 2**	**item 1**	**item 2**	**item 3**	**item 4**	**item 5**	**item 6**	**item 7**
Nature	1.60	1.90	1.64	0.42	**2.69**	**3.04**	-0.43
God	0.85	**2.78**	**2.69**	0.66	**2.00**	1.10	1.54
Institutions	0.83	**2.74**	**1.98**	**4.26**	**3.89**	**2.87**	-1.22
Other People	1.37	1.81	1.73	**2.87**	**4.76**	1.23	**-2.36**
Yourself	-0.18	-0.85	-1.10	0.88	-1.80	-1.29	-1.29
Chance	0.47	1.32	0.89	0.67	1.92	0.72	**-2.03**

Table: T-values of paired sample t-tests per item and actor (for Others minus for Yourself), to compare means for the two perspectives. Values in bold are significant at 5% level (two-sided, critical values are ±1.96).

Remarks:

- There are interesting patterns of large t-values (both positive and negative), which are quite different for the two studies. Interpretation is left to the experts ;-)
- Several methods have been proposed to control the family wise error rate in case of multiple comparisons. The simple and conservative Bonferroni correction implies replacing alpha by alpha/42 in our case. This results in critical values ±3.24 and yields 3 significant t-values for study 1, and also 3 significant t-values for study 2. The Bonferroni-Holm procedure (less conservative) orders the t-values and starts the largest in magnitude and alpha/42, where 42 is decreased by one for each null hypothesis that is rejected. This procedure also results in 3 significant t-values for both studies. We conclude that overall the mean differences between the two perspectives are rather small with only 3 out of 42 t-values being significant in both studies.

T-tests to compare means for the two studies

We do a two-sample t-test for each item and actor and perspective separately. We assume that the variances are equal in the two samples (but this is questionable; see the table on page 1). Hence, we obtain 84 t-values in total.

**for Others**	**item 1**	**item 2**	**item 3**	**item 4**	**item 5**	**item 6**	**item 7**
**Nature**	0.83	0.77	0.70	1.30	0.75	0.33	0.27
**God**	**-2.96**	**-3.11**	**-3.61**	**-2.41**	**-3.08**	**-2.90**	**-2.86**
**Institutions**	0.59	0.35	0.86	0.10	-0.08	0.12	-0.69
**Other People**	1.10	0.38	1.75	1.85	0.86	1.45	-0.54
**Yourself**	**2.04**	1.73	**2.40**	**2.53**	1.77	1.61	1.36
**Chance**	0.79	-0.44	0.17	1.14	-0.23	0.20	0.49
**for Yourself**	**item 1**	**item 2**	**item 3**	**item 4**	**item 5**	**item 6**	**item 7**
**Nature**	1.26	1.39	1.30	0.61	1.74	**2.34**	**1.99**
**God**	**-2.45**	**-2.09**	**-2.34**	**-2.14**	**-2.16**	**-2.20**	-1.78
**Institutions**	1.72	**2.37**	1.78	1.80	1.06	1.92	**2.32**
**Other People**	**2.96**	**3.61**	**2.28**	**3.28**	1.51	1.92	**2.14**
**Yourself**	0.43	0.39	1.68	1.94	**2.30**	**2.35**	**3.17**
**Chance**	0.23	-0.39	-0.97	-0.22	-0.01	0.28	0.85

Table: T-values of two-sample t-tests per item and actor and perspective (study 1 minus study 2), to compare means for the two studies. Values in bold are significant at 5% level (two-sided, critical values are ±1.96).

Remarks:

- There are interesting patterns of large t-values (both positive and negative), which are quite similar for the two perspectives. Interpretation is left to the experts ;-)
- The Bonferroni correction implies replacing alpha by alpha/84 in our case, which results in critical values ±3.43 and yields 1 significant t-value for the Others perspective, and also 1 significant t-value for the Yourself perspective. The Bonferroni-Holm procedure also results in 1 significant t-value for both perspectives. We conclude that overall the mean differences between the two studies are rather small with only 2 out of 84 t-values being significant.

T-tests to compare means for the order of questions in the questionnaire (counterbalancing)

In each study, the order of the questions (order=1 for first Others and then Yourself, order=2 for first Yourself and then Others) is varied. The numbers of subjects (without missing data) with the same order of questions are as follows:

study 1:	61 with order=1,	48 with order=2
study 2:	118 with order=1,	118 with order=2

We do a two-sample t-test for each item and actor and perspective and study. For study 1, the t-values are given in the table below.

**for Others**	**item 1**	**item 2**	**item 3**	**item 4**	**item 5**	**item 6**	**item 7**
**Nature**	0.79	-1.30	-1.20	-1.81	-1.19	-1.69	-0.33
**God**	-0.85	-0.83	-1.47	-0.87	-0.77	-1.57	-0.16
**Institutions**	-0.29	-0.30	**2.26**	-0.71	**-1.97**	-0.96	-1.08
**Other People**	**-2.12**	-0.49	-0.31	-0.56	-1.05	-1.06	-1.70
**Yourself**	-1.14	-1.26	0.58	0.90	-1.08	-1.05	-1.92
**Chance**	-0.50	-1.23	-1.26	0.28	-1.29	-1.95	-1.06
**for Yourself**	**item 1**	**item 2**	**item 3**	**item 4**	**item 5**	**item 6**	**item 7**
**Nature**	**-2.14**	**-2.33**	-1.46	-0.86	-1.61	-0.88	0.55
**God**	-0.39	-0.92	-0.92	-0.77	-0.15	-0.42	-0.06
**Institutions**	-0.09	-0.06	-0.65	-0.31	1.27	0.84	0.74
**Other People**	0.56	0.73	-0.18	0.01	0.82	0.79	-0.34
**Yourself**	-0.38	-0.38	0.27	-0.55	-1.12	-1.45	-0.77
**Chance**	-1.13	-0.54	-1.19	0.63	-1.38	-0.79	-0.46

Table: T-values of two-sample t-tests per item and actor and perspective (order=1 minus order=2), to compare means for the orders of questions for study 1. Values in bold are significant at 5% level (two-sided, critical values are ±1.96).

Remark:

- The Bonferroni correction (critical values ±3.43) and the Bonferroni-Holmes procedure both yield no significant t-values out of 84. We conclude that overall the mean differences between the two orders are not significant for study 1.

For study 2, the t-values are given in the table below.

**for Others**	**item 1**	**item 2**	**item 3**	**item 4**	**item 5**	**item 6**	**item 7**
**Nature**	0.70	1.02	0.63	0.50	-0.36	0.45	0.42
**God**	**-2.37**	-1.29	-1.30	-1.32	-0.91	-1.05	-0.41
**Institutions**	0.83	0.38	1.11	0.69	-0.44	0.58	0.40
**Other People**	0.48	1.31	0.97	0.10	-0.09	0.27	0.52
**Yourself**	-1.08	-2.11	-0.54	0.29	-0.58	0.33	-0.90
**Chance**	1.78	1.99	1.05	**2.32**	1.59	**2.10**	-0.29
**for Yourself**	**item 1**	**item 2**	**item 3**	**item 4**	**item 5**	**item 6**	**item 7**
**Nature**	-1.21	0.15	0.43	-0.14	0.50	0.87	0.45
**God**	-0.54	-0.51	-0.26	-0.57	-0.14	-0.66	-1.03
**Institutions**	-1.62	0.09	0.22	0.60	0.33	0.34	0.35
**Other People**	0.59	-0.40	1.07	0.90	1.42	0.66	0.92
**Yourself**	1.47	**2.42**	1.42	1.26	0.99	0.90	1.43
**Chance**	0.87	0.77	0.70	1.23	0.73	1.85	**2.74**

Table: T-values of two-sample t-tests per item and actor and perspective (order=1 minus order=2), to compare means for the orders of questions for study 2. Values in bold are significant at 5% level (two-sided, critical values are ±1.96).

Remark:

- The Bonferroni correction (critical values ±3.43) and the Bonferroni-Holmes procedure both yield no significant t-values out of 84. We conclude that overall the mean differences between the two orders are not significant for study 2.

Principal Component Analysis (PCA) for the perspectives and studies separately

For each study and each perspective separately, we do a PCA with Varimax rotation (resulting in (hopefully) interpretable orthogonal components) on the correlation matrix with 42 items (7 items for each of the 6 actors). Below, we present for each

PCA the SPSS table with explained variances, and a table with rotated loadings. We use 5 components in each PCA. Adding a sixth component yields either a non-interpretable component or a component with small loadings (around 0.4).

Total Variance Explained[a]

Component	Extraction Sums of Squared Loadings			Rotation Sums of Squared Loadings		
	Total	% of Variance	Cumulative %	Total	% of Variance	Cumulative %
1	11,565	27,535	27,535	7,851	18,693	18,693
2	8,105	19,297	46,833	6,618	15,758	34,451
3	4,370	10,404	57,237	5,543	13,198	47,650
4	3,484	8,296	65,533	4,915	11,702	59,352
5	2,145	5,107	70,640	4,741	11,288	70,640

Extraction Method: Principal Component Analysis.

a. Only cases for which Study = US lab data (run in the lab) are used in the analysis phase. Perspective: for Others.

Total Variance Explained[a]

Component	Extraction Sums of Squared Loadings			Rotation Sums of Squared Loadings		
	Total	% of Variance	Cumulative %	Total	% of Variance	Cumulative %
1	11,594	27,605	27,605	8,000	19,047	19,047
2	7,662	18,242	45,847	6,868	16,353	35,400
3	5,236	12,467	58,314	5,574	13,272	48,672
4	3,839	9,139	67,453	5,476	13,038	61,710
5	2,515	5,987	73,440	4,927	11,731	73,440

Extraction Method: Principal Component Analysis.

a. Only cases for which Study = US lab data (run in the lab) are used in the analysis phase. Perspective: for Yourself.

Total Variance Explained[a]

Component	Extraction Sums of Squared Loadings			Rotation Sums of Squared Loadings		
	Total	% of Variance	Cumulative %	Total	% of Variance	Cumulative %
1	13,290	31,643	31,643	8,551	20,359	20,359
2	7,544	17,961	49,604	6,488	15,448	35,806
3	4,723	11,245	60,850	5,609	13,355	49,161
4	3,337	7,944	68,794	5,414	12,891	62,052
5	2,141	5,096	73,890	4,972	11,838	73,890

Extraction Method: Principal Component Analysis.

a. Only cases for which Study = M-turk data (run online) are used in the analysis phase. Perspective: for Others.

Total Variance Explained[a]

Component	Extraction Sums of Squared Loadings			Rotation Sums of Squared Loadings		
	Total	% of Variance	Cumulative %	Total	% of Variance	Cumulative %
1	12,617	30,040	30,040	9,044	21,533	21,533
2	7,651	18,218	48,257	6,466	15,394	36,927
3	5,290	12,596	60,853	5,637	13,420	50,347

4	3,691	8,789	69,643	5,442	12,957	63,304
5	2,418	5,756	75,399	5,080	12,095	75,399

Extraction Method: Principal Component Analysis.

a. Only cases for which Study = M-turk data (run online) are used in the analysis phase. Perspective: for Yourself.

Rotated Component Matrix[a,b]

	Component				
	1	2	3	4	5
item1	,249	,188	,739	,132	,101
	-,030	,908	,177	-,071	,066
	,741	,028	-,015	,136	,029
	,667	,071	,027	,263	,149
	,010	-,046	,061	,093	,777
	,001	-,107	,116	,853	,038
item 2	,038	,305	,775	,093	,132
	-,048	,949	,166	-,102	-,039
	,762	,023	,024	,101	,094
	,727	-,163	,143	,275	,129
	,175	-,046	,094	,151	,786
	,118	-,177	,064	,806	,139
item 3	,152	,222	,832	,180	,103
	,033	,938	,222	-,072	-,030
	,646	,049	,031	-,003	,040
	,703	-,050	,104	,128	,269
	,070	,049	,101	,057	,836
	,109	-,089	,056	,851	,077
item 4	,157	,216	,814	,212	,036
	-,054	,965	,165	-,089	,000
	,580	-,024	,008	,158	,108
	,715	-,149	-,027	,352	,082
	,153	-,061	,072	,016	,806
	,129	-,068	,150	,787	-,193
item 5	,079	,219	,882	,170	,097
	-,067	,928	,197	-,125	-,017
	,803	,019	,135	,017	,039
	,681	-,167	,265	,146	,176
	,263	,053	,034	-,022	,778
	,253	-,109	,188	,817	,052
item 6	,191	,190	,882	,140	,139
	,015	,934	,237	-,049	-,011
	,784	,003	,213	-,025	,081
	,736	-,180	,197	,137	,181
	,297	,021	,141	,035	,798
	,155	-,045	,284	,815	,104
item 7	,166	,176	,725	,069	,170
	-,092	,886	,202	-,174	-,026
	,714	,136	,162	-,211	,124
	,688	-,039	,221	-,086	,175
	,306	,009	,318	-,105	,688
	,316	-,031	,313	,387	,121

Extraction Method: Principal Component Analysis.
Rotation Method: Varimax with Kaiser Normalization.

a. Rotation converged in 6 iterations.

b. Only cases for which Study = US lab data (run in the lab) are used in the analysis phase. Perspective: for Others.

<u>Study 1, for Others</u>

comp 1 = Institutions & Other People
comp 2 = God
comp 3 = Nature
comp 4 = Chance
comp 5 = Yourself

eigenvalue comp 6 = 1.57

Rotated Component Matrix[a,b]

	Component				
	1	2	3	4	5
item 1	,160	,063	,859	,048	-,009
	-,031	,948	,085	-,136	-,066
	,757	-,104	,108	-,066	-,065
	,713	-,168	-,074	-,002	-,023
	-,054	,067	-,085	,082	,781
	,187	-,180	,144	,835	-,061
item 2	,084	,195	,875	,096	,079
	-,007	,951	,112	-,099	,010
	,731	-,013	,112	-,132	,082
	,617	-,286	-,080	,051	,147
	,129	,002	-,034	,137	,735
	,035	-,128	,071	,826	,058
item 3	-,010	,105	,877	,230	,079
	-,089	,946	,136	-,126	-,071
	,696	,004	,114	,179	,139
	,701	-,180	,016	,385	,197
	,189	,003	,164	-,005	,757
	,143	-,080	,313	,836	,018
item 4	-,028	,086	,833	,272	-,022
	-,108	,957	,099	-,112	-,045
	,613	,010	,287	,038	-,014
	,719	-,236	,036	,119	,063
	,248	-,044	,060	,070	,834
	,211	-,188	,113	,769	-,016
item 5	,104	,117	,875	,173	,109
	-,081	,945	,114	-,137	-,051
	,761	,180	,229	,215	,090
	,690	,044	,122	,311	,113
	,146	-,178	-,033	,014	,834
	,115	-,192	,236	,859	,028
item 6	,058	,166	,864	,282	,052
	-,074	,960	,121	-,123	-,028
	,779	,175	,115	,238	,160
	,735	-,041	,021	,321	,283
	,171	-,071	,121	-,046	,854
	,099	-,026	,261	,851	,176
item 7	,284	,038	,627	,146	,057
	,013	,934	,077	-,074	,004
	,837	,103	,044	,060	,243
	,777	-,037	-,086	,110	,223
	,160	-,033	,152	-,007	,811
	,391	-,063	,245	,638	,103

Extraction Method: Principal Component Analysis.
Rotation Method: Varimax with Kaiser Normalization.

a. Rotation converged in 6 iterations.

b. Only cases for which Study = US lab data (run in the lab) are used in the analysis phase. Perspective: for Yourself.

<u>Study 1, for Yourself</u>

comp 1 = Institutions & Other People

comp 2 = God

comp 3 = Nature

comp 4 = Chance

comp 5 = Yourself

eigenvalue comp 6 = 1.29

Rotated Component Matrix[a,b]

	Component				
	1	2	3	4	5
item 1	,196	,163	,102	,818	,175
	,018	,942	-,026	,179	-,076
	,723	,007	,158	,156	,172
	,710	-,078	,139	,196	,189
	,240	,042	,793	,095	,059
	,120	-,113	,085	,110	,815
item 2	,189	,220	,077	,823	,134
	,025	,936	-,043	,175	-,078
	,777	,065	,089	,032	,070
	,751	-,080	,009	,047	,135
	,155	-,110	,790	,087	,121
	,072	-,107	,041	,143	,859
item 3	,143	,222	,077	,836	,195
	,008	,941	-,041	,177	-,076
	,805	,086	,163	,002	,088
	,778	-,065	,118	,097	,057
	,265	-,004	,838	,080	,092
	,070	-,124	,100	,153	,888
item 4	,186	,184	,066	,829	,239
	,013	,903	-,042	,204	-,074
	,766	,013	,082	,060	,145
	,783	-,079	,124	,110	,103
	,237	-,055	,843	,061	,156
	,151	-,075	,053	,099	,843
item 5	,159	,280	,086	,781	,177
	-,020	,941	-,057	,152	-,083
	,744	,137	,225	,181	,036
	,757	-,012	,205	,217	,067
	,230	-,070	,870	,068	,064
	,071	-,109	,064	,186	,892
item 6	,162	,216	,115	,840	,188
	,017	,932	,014	,169	-,083
	,806	,077	,223	,155	,005
	,737	-,045	,253	,242	-,004
	,270	-,049	,868	,075	,046
	,149	,005	,211	,226	,790
item 7	,259	,095	,126	,727	,047
	,016	,901	-,016	,135	-,103
	,630	,144	,327	,185	-,009
	,650	,040	,343	,157	-,016
	,217	,022	,819	,133	,128
	,244	-,025	,276	,337	,424

Extraction Method: Principal Component Analysis.
Rotation Method: Varimax with Kaiser Normalization.

a. Rotation converged in 6 iterations.

b. Only cases for which Study = M-turk data (run online) are used in the analysis phase. Perspective: for Others.

Study 2, for Others

comp 1 = Institutions & Other People

comp 2 = God

comp 3 = Yourself

comp 4 = Nature

comp 5 = Chance

eigenvalue comp 6 = 1.02

Rotated Component Matrix[a,b]

	Component				
	1	2	3	4	5
item 1	,138	,161	,793	,107	,272
	-,013	,936	,167	-,065	-,047
	,758	,072	,037	,133	,175
	,714	,015	,203	,062	,132
	,120	-,040	-,009	,869	,090
	,094	-,095	,145	,143	,835
item 2	,188	,186	,870	,135	,142
	,014	,933	,124	-,086	-,107
	,847	,086	,021	,100	,042
	,776	,028	,135	-,009	-,031
	,085	-,031	,085	,791	,123
	-,014	-,061	,103	,082	,875
item 3	,132	,128	,875	,065	,188
	,011	,940	,153	-,094	-,072
	,818	,032	,000	,126	,137
	,762	-,067	,201	,036	,086
	,172	-,119	,121	,856	,038
	,023	-,104	,166	,059	,885
item 4	,179	,169	,797	,097	,251
	-,006	,934	,126	-,104	-,043
	,782	-,016	,039	,076	,202
	,747	-,023	,095	,084	,229
	,159	-,087	,134	,869	,142
	,120	-,110	,111	,130	,849
item 5	,141	,182	,837	,059	,222
	,002	,951	,114	-,083	-,094
	,762	,111	,188	,138	-,020
	,726	-,006	,310	,119	,060
	,183	-,171	,033	,853	,167
	,087	-,133	,187	,088	,884
item 6	,185	,157	,864	,096	,113
	,048	,943	,134	-,086	-,097
	,842	,020	,068	,143	,016
	,817	-,093	,158	,177	,032
	,206	-,078	,121	,869	,031
	,180	-,057	,309	,150	,728
item 7	,297	,075	,756	,076	,014
	,075	,910	,097	-,077	-,146
	,787	,024	,083	,123	-,094
	,743	-,027	,159	,194	-,102
	,169	-,068	,126	,800	,139
	,252	-,098	,359	,246	,444

Extraction Method: Principal Component Analysis.
Rotation Method: Varimax with Kaiser Normalization.

a. Rotation converged in 6 iterations.

b. Only cases for which Study = M-turk data (run online) are used in the analysis phase. Perspective: for Yourself.

Study 2, for Yourself

comp 1 = Institutions & Other People
comp 2 = God
comp 3 = Nature
comp 4 = Yourself
comp 5 = Chance

eigenvalue comp 6 = 1.13

Remarks:

- The explained variances of the PCAs with 5 components are good: 70-75 percent.
- After rotation, the five components have a very clear interpretation: each component is interpreted as one actor, except for Human Institutions and Other People. The latter are found combined in one factor. This reflects the high correlations between these two actors and the low correlations between the other actors.
- The loadings of some components are less pronounced for item 7, which correlates the least with other items.
- The eigenvalues of the sixth components are around 1-1.5, while the eigenvalues of the fifth components are around 2.1-2.5. Hence, also the eigenvalue larger than 1 criterion indicates that we should include 5 (or 6) components for each PCA.

Principal Component Analysis for both studies together, for the perspectives separately

Next, we combine both studies and do a PCA with Varimax rotation for each perspective separately. Below, the results are presented analogous to the above. Not surprisingly, the conclusions are the same as for the PCAs of the two studies separately.

Total Variance Explained

Component	Extraction Sums of Squared Loadings			Rotation Sums of Squared Loadings		
	Total	% of Variance	Cumulative %	Total	% of Variance	Cumulative %
1	12,734	30,319	30,319	8,343	19,863	19,863
2	7,662	18,244	48,563	6,512	15,505	35,368
3	4,568	10,877	59,440	5,393	12,841	48,209
4	3,357	7,992	67,432	5,313	12,649	60,857
5	2,150	5,120	72,552	4,912	11,695	72,552

Extraction Method: Principal Component Analysis. Perspective: for Others.

Total Variance Explained

Component	Extraction Sums of Squared Loadings			Rotation Sums of Squared Loadings		
	Total	% of Variance	Cumulative %	Total	% of Variance	Cumulative %

1	12,334	29,367	29,367	8,872	21,125	21,125
2	7,519	17,903	47,270	6,524	15,534	36,659
3	5,161	12,288	59,558	5,548	13,210	49,869
4	3,805	9,059	68,617	5,235	12,464	62,332
5	2,421	5,764	74,381	5,061	12,049	74,381

Extraction Method: Principal Component Analysis. Perspective: for Yourself.

Rotated Component Matrix[a]

	Component				
	1	2	3	4	5
item 1	,216	,157	,803	,100	,159
	,002	,933	,176	-,013	-,074
	,731	,007	,111	,119	,160
	,699	-,049	,155	,136	,200
	,183	,007	,088	,789	,066
	,094	-,116	,111	,071	,821
item 2	,147	,234	,815	,098	,122
	,000	,942	,169	-,050	-,083
	,773	,050	,033	,087	,072
	,742	-,106	,078	,037	,166
	,165	-,101	,092	,785	,128
	,087	-,119	,116	,058	,845
item 3	,149	,210	,842	,086	,187
	,011	,942	,182	-,049	-,073
	,763	,065	,021	,126	,051
	,755	-,075	,107	,160	,065
	,219	-,005	,090	,838	,080
	,081	-,112	,121	,093	,883
item 4	,179	,176	,830	,065	,228
	-,009	,922	,192	-,036	-,078
	,716	,002	,047	,081	,141
	,764	-,119	,070	,108	,171
	,215	-,066	,062	,841	,124
	,149	-,079	,111	-,004	,829
item 5	,141	,254	,814	,093	,171
	-,037	,940	,160	-,053	-,093
	,765	,111	,154	,169	,038
	,734	-,056	,217	,195	,097
	,238	-,043	,060	,848	,043
	,118	-,100	,176	,060	,878
item 6	,175	,207	,850	,123	,176
	,014	,935	,183	-,002	-,072
	,802	,063	,160	,183	-,001
	,743	-,087	,216	,230	,041
	,280	-,036	,094	,847	,042
	,156	-,005	,235	,181	,801
item 7	,234	,120	,724	,138	,056
	-,019	,903	,149	-,024	-,120
	,655	,160	,160	,267	-,056
	,662	,036	,159	,289	-,028
	,243	,022	,175	,792	,073
	,269	-,020	,316	,233	,423

Extraction Method: Principal Component Analysis.
Rotation Method: Varimax with Kaiser Normalization. Perspective: for Others.

a. Rotation converged in 6 iterations.

Study 1+2, for Others

comp 1 = Institutions & Other People
comp 2 = God
comp 3 = Nature
comp 4 = Yourself
comp 5 = Chance

eigenvalue comp 6 = 1.12

Rotated Component Matrix[a]

	Component				
	1	2	3	4	5
item 1	,147	,128	,813	,079	,213
	-,029	,941	,138	-,070	-,066
	,754	,026	,049	,084	,123
	,721	-,045	,131	,046	,087
	,078	-,007	-,023	,848	,083
	,126	-,118	,143	,087	,839
item 2	,167	,185	,870	,123	,130
	,000	,940	,115	-,066	-,102
	,814	,059	,037	,100	,002
	,751	-,058	,089	,029	-,025
	,103	-,021	,048	,774	,125
	,003	-,073	,087	,075	,869
item 3	,104	,115	,882	,069	,191
	-,026	,944	,144	-,092	-,084
	,788	,020	,033	,126	,138
	,761	-,104	,153	,073	,146
	,183	-,088	,129	,831	,033
	,047	-,088	,194	,046	,879
item 4	,129	,146	,808	,062	,259
	-,042	,943	,115	-,092	-,061
	,732	-,006	,104	,056	,158
	,745	-,087	,086	,082	,190
	,190	-,084	,120	,859	,119
	,143	-,122	,103	,092	,839
item 5	,140	,151	,857	,074	,197
	-,030	,952	,110	-,078	-,103
	,763	,124	,203	,116	,034
	,728	,003	,261	,113	,109
	,185	-,176	,017	,848	,126
	,099	-,142	,193	,071	,885
item 6	,167	,141	,876	,084	,144
	,004	,952	,127	-,074	-,101
	,828	,055	,083	,145	,060
	,805	-,082	,123	,199	,093
	,209	-,082	,121	,862	,013
	,170	-,043	,292	,154	,761
item 7	,309	,052	,716	,070	,045
	,051	,919	,089	-,060	-,126
	,804	,038	,069	,155	-,061
	,763	-,031	,083	,200	-,047
	,181	-,072	,137	,801	,096
	,304	-,091	,317	,198	,503

Extraction Method: Principal Component Analysis.
Rotation Method: Varimax with Kaiser Normalization. Perspective: for Yourself.

a. Rotation converged in 6 iterations.

<u>Study 1+2, for Yourself</u>

comp 1 = Institutions & Other People
comp 2 = God
comp 3 = Nature
comp 4 = Yourself
comp 5 = Chance

eigenvalue comp 6 = 1.10

3-way Candecomp/Parafac (CP) analysis per perspective

As an alternative to PCA per perspective as above, we can also fit the 3-way CP model to the 345×7×6 arrays for each perspective. For more details on CP, see my previous report. The CP analysis is done such that the results can be compared to the PCAs above. In matrix notation, PCA per perspective can be written as $\mathbf{X} \approx \mathbf{AL}^T$, where **X** is the 345×42 data matrix (rows are subjects, columns are items for one perspective), **A** is the 345×5 matrix with the component scores for the first five PCs, and **L** is the 42×5 matrix of loadings of the 42 items on the 5 PCs. Note that the columns of **X** are standardized in the PCAs above. When the columns of **X** are only centered, the results are analogous. The columns of **A** are orthogonal, as usual in PCA.

The Varimax rotation of the PCs can be written as $\mathbf{X} \approx \mathbf{AL}^T = (\mathbf{AQ})(\mathbf{LQ})^T$, where **Q** is a 5×5 orthonormal rotation matrix. The Varimax algorithms finds **Q** such that the new loadings **LQ** have maximal variance of the squared loadings per column. This implies that (usually, hopefully) each column of **LQ** has a small number of large loadings and a lot of small ones, thus making interpretation of the PCs easier. As we have seen above, this works excellent in the PCAs per perspective.

In a 3-way CP analysis of **X** (with 5 components), the 42 items are explicitly considered as 6 groups of 7 items. The loadings **L** are written as (**B•C**), with **B** a 7×5 matrix of loadings of the 7 items on the 5 components, and **C** a 6×5 matrix of loadings of the 6 actors on the 5 components. Formally, in 3-way CP the loading (i,r) of **L** is replaced by the product of loading (j,r) of **B** multiplied by loading (k,r) of **C**, where item i (of 42 in total) corresponds to item j (of 7 in total) and actor k. A 3-way CP solution (**A**,**B**,**C**) is unique (under some conditions, which hold in our case) and cannot be rotated as in PCA. It is interesting to see if the CP loadings for the actors in **B** are of the same well interpretable form as those in **L** in the PCAs above (for each of the 7 items separately).

The matrices **B** and **C** of the 3-way CP solutions per perspective are given in the table below. (Details: columns of X are centered (across mode A), no normalization, R=5 components, orthogonality in mode A, 10 runs with random starting values, ALS algorithm, convergence criterion 1e-9, scaling of **B** and **C** such that mean squared loading equals 1 per column.) The explained variance equals 75.44 percent for Others, and 76.81 percent for Yourself. As can be seen, the item loadings are around 1.00 with some smaller loadings for item 7. The actor loadings are not as nice as in the PCAs above. Some components are contrasts in terms of the actors (i.e., with both large positive and large negative actor loadings for the same component). Some components have large positive actor loadings for more than two actors. Note that Human Institutions and Other People tend to have similar loadings for each component. When comparing the CP solutions for the two perspectives, it can be seen that components 1 and 2 for Others are similar to components 2 and 1 for Yourself, respectively. Also, component 4 is similar in the two solutions.

For each perspective, we also constructed a 3-way CP solution from the corresponding PCA above. That is, we fit an approximation **L** ≈ (**B•C**) to the PCA loading matrix **L**. Here, matrix **B** has the same well interpretable form as in the PCA for each perspective. For these 3-way CP solutions, the explained variance equals 74.91 percent for Others and 76.28 percent for Yourself. Hence, in terms of fit, these 3-way CP solutions are very close to the ones obtained by fitting the 3-way CP model.

We tried to obtain these solutions by including constraints (nonnegativity, sparsity) in the actors mode of the 3-way CP model, but this approach was not successful. (Idea: perhaps include the Varimax objective in the estimation of **B**?)

for Others	comp 1	comp 2	comp 3	comp 4	comp 5
item 1	0.93	0.96	0.92	1.01	0.90
item 2	0.89	1.03	1.03	1.06	1.07
item 3	0.95	0.99	1.03	1.05	1.08
item 4	0.92	0.97	1.03	1.13	0.98
item 5	1.04	1.05	1.07	1.12	1.06
item 6	1.11	1.00	1.01	0.96	1.07
item 7	1.12	1.01	0.91	0.55	0.80
Nature	1.23	-0.25	1.60	0.14	-1.05
God	0.50	2.39	1.51	-0.17	-0.51
Institutions	1.05	-0.02	0.03	0.47	1.31
Other People	1.03	-0.24	-0.03	0.52	1.14
Yourself	1.30	-0.05	-1.06	-0.46	-0.76
Chance	0.62	-0.42	-0.10	2.29	-1.03
% explained var	24.21	20.20	14.40	8.92	7.71

Table: Perspective: for Others. Matrices **B** of item loadings and **C** of actor loadings for the 3-way CP solution with R=5 components and orthogonality in the subjects mode, for the 345×7×6 data array. Total explained variance equals 75.44 percent.

for Yourself	comp 1	comp 2	comp 3	comp 4	comp 5
item 1	0.98	0.90	0.99	1.03	1.14
item 2	1.01	0.89	1.03	1.08	1.03
item 3	0.96	0.98	1.10	1.09	0.92
item 4	0.97	0.99	1.00	1.08	1.01
item 5	1.06	1.06	1.01	1.11	1.00
item 6	1.01	1.08	1.03	0.93	0.95
item 7	1.01	1.08	0.82	0.57	0.93
Nature	0.17	1.11	1.96	0.33	-0.55
God	2.33	-0.35	1.17	0.07	0.74
Institutions	0.45	1.21	-0.62	0.09	-0.51
Other People	0.28	1.22	-0.55	0.12	-0.58
Yourself	-0.34	1.09	-0.04	-0.22	2.13
Chance	-0.37	0.71	0.32	2.41	0.16

% explained var	23.75	23.16	12.86	9.21	7.83

Table: Perspective: for Yourself. Matrices **B** of item loadings and **C** of actor loadings for the 3-way CP solution with R=5 components and orthogonality in the subjects mode, for the 345×7×6 data array. Total explained variance equals 76.81 percent.

4-way Candecomp/Parafac (CP) analysis on the complete dataset

Here, we consider the complete dataset of 84 items (both perspectives together). Doing a PCA (and Varimax rotation) on the 345×84 data matrix results in similar loadings as in the PCAs for the perspectives separately. Hence, we have $\mathbf{X} \approx \mathbf{AL}^T$, where **X** is the 345×84 data matrix, **A** is the 345×5 matrix with the component scores for the first five PCs, and **L** is the 84×5 matrix of loadings of the 84 items on the 5 PCs. As in the PCAs above, each component has a clear interpretation in terms of the actors (each actor is represented by one component, and Human Institutions and Other People share a component; results not reported).

In a 4-way CP analysis of **X** (with 5 components), the 84 items are explicitly considered as 6 groups of 7 items for 2 perspectives. The loadings **L** are written as (**B•C•D**), with **B** a 7×5 matrix of loadings of the 7 items on the 5 components, **C** a 6×5 matrix of loadings of the 6 actors on the 5 components, and **D** a 2×5 matrix of loadings of the 2 perspectives on the 5 components. Formally, in 4-way CP the loading (i,r) of **L** is replaced by the product of loading (j,r) of **B** multiplied by loading (k,r) of **C** multiplied by loading (l,r) of **D**, where item i (of 84 in total) corresponds to item j (of 7 in total), actor k, and perspective l. A 4-way CP solution (**A**,**B**,**C**,**D**) is unique (under some conditions, which hold in our case) and cannot be rotated as in PCA. It is interesting to see if the CP loadings for the actors in **B** are of the same well interpretable form as those in **L** in the PCA on the 84 items (for each of the 7 items and each perspective separately).

The matrices **B**, **C**, and **D** of the 4-way CP solution are given in the table below. (Details: columns of X are centered (across mode A), no normalization, R=5 components, orthogonality in mode A, 10 runs with random starting values, ALS algorithm, convergence criterion 1e-9, scaling of **B** and **C** and **D** such that mean squared loading equals 1 per column.) The explained variance equals 69.31 percent. Components 1,2,4 are similar to those found in the 3-way CP solutions above. The item loadings are all around 1.00 except for a smaller loading for item 7 on component 4. The actor loadings are not as nice as in the PCA, with components 3 and 5 being contrasts, and components 1 and 2 having large positive loadings of more than one actor (and not the pair Human Institutions and Other People).

We also constructed a 4-way CP solution from the PCA on all 84 items. That is, we fit an approximation **L** ≈ (**B•C•D**) to the PCA loading matrix **L**. Here, matrix **B** has the same well interpretable form as in the PCA. For this 4-way CP solution, the explained variance equals 68.60 percent. Hence, in terms of fit, this 4-way CP solution is very close to the one obtained by fitting the 4-way CP model.

	comp 1	comp 2	comp 3	comp 4	comp 5
**item 1**	0.97	0.93	0.94	0.99	0.98
**item 2**	1.03	0.89	0.97	1.05	1.02
**item 3**	1.02	0.95	1.00	1.07	0.99
**item 4**	1.00	0.95	0.98	1.09	0.99
**item 5**	1.04	1.03	1.07	1.13	1.04
**item 6**	1.01	1.12	1.02	0.97	1.01
**item 7**	0.92	1.10	1.02	0.61	0.97
**Nature**	1.14	0.92	1.49	0.39	0.53
**God**	2.09	0.57	-1.76	0.02	0.50
**Institutions**	-0.26	1.03	0.10	-0.00	1.50
**Other People**	-0.34	0.96	0.30	0.09	1.44
**Yourself**	-0.38	1.56	0.28	-0.27	-1.07
**Chance**	-0.16	0.65	0.72	2.40	-0.03
**for Others**	1.00	1.02	0.91	0.99	0.82
**for Yourself**	1.00	0.98	1.08	1.01	1.15
**% explained var**	22.48	18.53	12.81	8.17	7.32

Table: Matrices **B** of item loadings, **C** of actor loadings, and **D** of perspective loadings for the 4-way CP solution with R=5 components and orthogonality in the subjects mode, for the complete $345\times7\times6\times2$ data array. Total explained variance equals 69.31 percent.
