# Supplementary material for: Belief in a Just What? Demystifying Just World Beliefs by Distinguishing Sources of Justice
Source: PLoS One. 2015 Mar 24;10(3):e0120145. doi: 10.1371/journal.pone.0120145 (PMC4372594; doi:10.1371/journal.pone.0120145)
Supplement: S2 File — (PDF) [file pone.0120145.s006.pdf]

Supplementary analyses for Step 2 - assessing uni- or multi-dimensionality of the BJT5 via bifactor analyses

We conducted a confirmatory factor analysis for the full BJT5 scale with the lavaan-package in R (Rosseel, 2012). The first step was to fit a 5-factor model using 7 items to predict each BJT5 dimension. All factors were allowed to correlate, but no hierarchical structure was specified initially. Separate factor analyses were conducted for items targeting self and other. Initial model fit indices approached benchmark standards for self and others respectively, χ^2 (550) = 1524.54, p<.001, CFI = .93, TLI=.92, SRMR = 0.06 and χ^2 (550) = 1346.24, p<.001, CFI = .94, TLI=.93, SRMR = 0.06. Inspection of modification indices revealed (rather unsurprisingly) that some of the similarly worded items across dimensions had high residual covariance. Allowing these items to covary resulted in acceptable model fit, χ^2 (531) = 1140.80, p<.001, CFI = .96, TLI=.95, SRMR = 0.06 and χ^2 (534) = 1060.36, p<.001, CFI = .96, TLI=.95, SRMR = 0.06.

Correlations between factors are displayed in Table S1. Correlations for items referring to self and others are listed separately, but as can be seen the differences between these are rather small. For ease of interpretation we draw your attention to the bottom third of the table where averaged correlations are listed. Correlations between Nature, Other people, Self and Chance all hovered around .30 (median = .33). But correlations with the factor referring to God were all markedly different. God correlated positively only with Nature, and showed a small negative correlation with Chance and (getting closer to zero) with Self and Other people.

Table S2.1. Correlations among factors of the BJT5, reordered so it becomes easier to see how correlations with God are different from correlations with other factors.

	God	Nature	Other people	Self
Self				
Nature	0.24			
Other people	-0.09	0.33		
Self	-0.18	0.23	0.39	
Chance	-0.19	0.40	0.27	0.25
Others				
Nature	0.37			
Other people	-0.06	0.37		
Self	-0.07	0.28	0.49	
Chance	-0.16	0.37	0.32	0.24
Average[1]				
Nature	0.31			
Other people	-0.07	0.35		
Self	-0.13	0.25	0.44	
Chance	-0.17	0.39	0.30	0.25

[1]Averages were computed by fisher-Z transforming correlations for self and other and inverse transforming the average.

Table S2.2 Factor loadings of bifactor models estimated in Step 2.

	Bifactor model		Modified bifactor model	
	Group factors	General factor	Group factor	Genera l factor
Self items				
Nature 1	0.65	0.51	0.63	0.53
Nature 2	0.78	0.53	0.77	0.54
Nature 7	0.67	0.56	0.68	0.56
God 1	0.96	0.01	0.94	-0.15
God 2	0.94	-0.01	0.93	-0.17
God 7	0.97	-0.01	0.95	-0.17
Other people 1	0.62	0.41	0.63	0.40
Other people 2	0.74	0.32	0.74	0.32
Other people 7	0.61	0.55	0.62	0.53
Self 1	0.77	0.31	0.78	0.30
Self 2	0.68	0.34	0.67	0.35
Self 7	0.70	0.47	0.69	0.47
Chance 1	0.72	0.44	0.68	0.50
Chance 2	0.82	0.32	0.79	0.38
Chance 7	0.54	0.63	0.51	0.64
Others				
Nature 1	0.70	0.55	0.66	0.57
Nature 2	0.71	0.45	0.75	0.44
Nature 7	0.43	0.60	0.45	0.58
God 1	0.93	0.12	0.94	-0.01
God 2	0.97	0.09	0.97	-0.05
God 7	0.90	0.11	0.90	-0.03
Other people 1	0.56	0.54	0.57	0.55
Other people 2	0.70	0.40	0.65	0.43
Other people 7	0.33	0.62	0.36	0.57
Self 1	0.68	0.44	0.69	0.43
Self 2	0.66	0.43	0.64	0.46
Self 7	0.54	0.58	0.55	0.55
Chance 1	0.77	0.27	0.72	0.36
Chance 2	0.82	0.28	0.80	0.36
Chance 7	0.25	0.65	0.17	0.67
