# Supplementary material for: Belief in a Just What? Demystifying Just World Beliefs by Distinguishing Sources of Justice
Source: PLoS One. 2015 Mar 24;10(3):e0120145. doi: 10.1371/journal.pone.0120145 (PMC4372594; doi:10.1371/journal.pone.0120145)
Supplement: S3 File — (PDF) [file pone.0120145.s007.pdf]

Supplementary materials: Scenarios in Sample 2

Nature/God – micro

Negative: You are collecting chestnuts in the park. A chestnut falls out of the tree on your head and injures you badly.

Positive: You are on a hike outside of town. Suddenly you see a beautiful bird sitting very close to you.

Nature/God - macro

Negative: The world is hit by storms and floods. Large parts of the world population lose their homes and possessions.

Positive: Weather conditions throughout the world are improving. The world population is enjoying the sun and profiting from the rain.

Other people - micro

Negative: You are out walking in the streets during the day. Suddenly you are verbally abused by a stranger.

Positive: You are in a queue in a shop. Suddenly someone pays you a compliment.

Other people – macro

Negative: Child abuse is prevalent across cultures worldwide. A large percentage of the world population is affected.

Positive: Online social media connect people all over the world with each other. The world population is more closely connected to each other than ever before.

Self – micro

Negative: You cross the road on your bike despite the traffic light

being red. You are hit by a car.

Positive: You are feeling incredibly happy and have a big smile on

your face. You notice that everyone you meet is influenced positively by this.

Self - macro

Negative: You put up a YouTube video showing people how to make

their own fireworks for New Year's Eve. Your video becomes a worldwide hit and everyone starts making their own fireworks. On New Year's Eve it turns out that those fireworks cause very serious injuries to many of the world population.

Positive: You discover that a simple herb combination cures a

serious mood disorder. People all over the world over benefit from your discovery.
