# Supplementary material for: Belief in a Just What? Demystifying Just World Beliefs by Distinguishing Sources of Justice
Source: PLoS One. 2015 Mar 24;10(3):e0120145. doi: 10.1371/journal.pone.0120145 (PMC4372594; doi:10.1371/journal.pone.0120145)
Supplement: S1 Table — (PDF) [file pone.0120145.s008.pdf]

S1_Table. Supplementary materials Step 5

Summary of hierarchical regression analyses examining effects of action type in the Nature versus Self scenarios (Step 5)

Predictor	Action type	M(SD) Nature scenario	M(SD) Self scenario	Nature scenario		Self scenario	
				β	*t-value*	*β*	*t-value*
	God	4.03(1.96)	3.69(2.09)				
Nature				-.109*	-2.56	-0.102*	-2.315
**God**				**0.808*****	**21.25**	**0.795*****	**20.17**
Other people				-.001	-0.20	0.048	1.215
Self				0.024	0.647	-0.036	-0.917
Chance				-.009	-0.234	0.042	1.00
R^2				0.612		0.583	
ΔR^2				0.606		0.577	
	Other people	5.15(1.19)	4.29(1.62)				
Nature				-0.09	-1.41	0.038	0.589
God				0.133*	2.244	0.305***	5.329
**Other people**				**0.223*****	**3.731**	**0.147***	**2.532**
Self				0.049	0.834	-0.039	-0.698
Chance				0.057	0.914	-0.021	-0.343
R^2				0.063		0.121	
ΔR^2				0.050		0.108	
	Self	3.58(1.96)	4.9(1.91)				
Nature				0.144	1.739	0.042	0.628
God				0.222***	3.792	0.202**	3.396
Other people				-0.28	-0.479	0.028	0.648
**Self**				**0.071**	**1.223**	**0.030**	**0.610**
Chance				0.033	0.533	0.009	0.887
R^2				0.083		0.050	
ΔR^2				0.070		0.035	
	No action	2.72(1.43)	2.54(1.73)				
Nature				0.158*	2.445	0.069	1.048
God				-0.044	-0.752	-0.050	-0.847
Other people				-0.052	-0.894	-0.055	-0.916
**Self**				**0.156****	**2.731**	**0.118***	**2.019**
Chance				0.140*	2.279	0.156*	2.488
R^2				0.101		0.062	
ΔR^2				0.088		0.048	

Note. N=345
* p<.05 ** p<.01 ***p<.001
